# Distinct roles of DNMT1-dependent and DNMT1-independent methylation patterns in the genome of mouse embryonic stem cells

**DOI:** 10.1186/s13059-015-0685-2

**Published:** 2015-06-02

**Authors:** Zhiguang Li, Hongzheng Dai, Suzanne N. Martos, Beisi Xu, Yang Gao, Teng Li, Guangjing Zhu, Dustin E. Schones, Zhibin Wang

**Affiliations:** Department of Environmental Health Sciences, Laboratory of Human Environmental Epigenomes, Bloomberg School of Public Health, Johns Hopkins University, 615 N. Wolfe Street, Room E7816, Baltimore, MD 21205 USA; Department of Diabetes Complications and Metabolism, Beckman Research Institute, City of Hope, Duarte, CA 91010 USA; Fenxian Central Hospital, 9588 Nanfeng Hwy, Fengxian District, Shanghai, 201406 China; The Sidney Kimmel Comprehensive Cancer Center and Department of Oncology, School of Medicine, Johns Hopkins University, Baltimore, MD 21205 USA

## Abstract

**Background:**

DNA methylation patterns are initiated by *de novo* DNA methyltransferases DNMT3a/3b adding methyl groups to CG dinucleotides in the hypomethylated genome of early embryos. These patterns are faithfully maintained by DNMT1 during DNA replication to ensure epigenetic inheritance across generations. However, this two-step model is based on limited data.

**Results:**

We generated base-resolution DNA methylomes for a series of DNMT knockout embryonic stem cells, with deep coverage at highly repetitive elements. We show that DNMT1 and DNMT3a/3b activities work complementarily and simultaneously to establish symmetric CG methylation and CHH (H = A, T or C) methylation. DNMT3a/3b can add methyl groups to daughter strands after each cycle of DNA replication. We also observe an unexpected division of labor between DNMT1 and DNMT3a/3b in suppressing retrotransposon long terminal repeats and long interspersed elements, respectively. Our data suggest that mammalian cells use a specific CG density threshold to predetermine methylation levels in wild-type cells and the magnitude of methylation reduction in DNMT knockout cells. Only genes with low CG density can be induced or, surprisingly, suppressed in the hypomethylated genome. Lastly, we do not find any association between gene body methylation and transcriptional activity.

**Conclusions:**

We show the concerted actions of DNMT enzymes in the establishment and maintenance of methylation patterns. The finding of distinct roles of DNMT1-dependent and -independent methylation patterns in genome stability and regulation of transcription provides new insights for understanding germ cell development, neuronal diversity, and transgenerational epigenetic inheritance and will help to develop next-generation DNMT inhibitors.

**Electronic supplementary material:**

The online version of this article (doi:10.1186/s13059-015-0685-2) contains supplementary material, which is available to authorized users.

## Background

DNA methylation patterns play key roles in the control of genomic imprinting, gene transcription, and genome stability [1–4]. Normal methylation patterns are essential for embryonic development and aberrant methylation patterns are associated with numerous human diseases, including many imprinting disorders and various types of cancer [3, 5, 6]. Cancer cells are characterized by global hypomethylation and promoter-specific hypermethylation, which have been implicated in carcinogenesis [7, 8]. Given the importance of DNA methylation patterns in human health, understanding how methylation patterns are set up and maintained by DNA methyltransferases (DNMTs) is of great importance. The standard model is that de novo methyltransferases DNMT3a/3b establish the methyl-CG landscape in the genome prior to implantation. Afterward, DNMT1 ensures the faithful copying of CG methylation from parental to daughter strand at replication forks [1, 2]. However, this ‘two-step’ model is based on limited data and new observations, such as CHH (H = A, T, and C) methylation, do not fit it [9]. Furthermore, in cells with impaired DNMT3a/3b but fully functional DNMT1, up to 30 % of CG sites in repeat regions of the mouse genome are hemimethylated [10]. This suggests that methylation inheritance mediated solely by DNMT1 is imperfect. Recent studies further show that DNMT1 may also have de novo methyltransferase activity [11, 12], underscoring the importance of refining the model by which DNA methylation patterns are established and maintained [13].

DNA methylation patterns are critical to maintain genome stability by repressing transposable elements (TEs) in the genome [14]; however, the mechanisms whereby methylation patterns repress TEs remain to be fully elucidated. About 40 % of mammalian genomes are composed of repetitive elements, including long interspersed nuclear elements (LINEs) and long terminal repeats (LTRs) [15]. LINE1 (L1) elements are still active in the human genome and L1-mediated retrotransposition events account for approximately 1 of every 1000 spontaneous disease-producing insertions in the human genome [16, 17]. Retrotransposition activity and related mutations are much higher in the mouse genome than the human genome (almost 100-fold difference) due to the fact that active LTRs exist in mouse but not in human [18]. While commonly associated with adverse effects, retrotransposition can also be beneficial to host cells. For example, L1 retrotransposition may contribute to neuronal diversity [19]. Loss of DNMT1 leads to a significant loss of methylation at intracisternal A-particle (IAP)-related elements and extensive transcription of IAPs in mouse embryos [20]. Despite their importance, it remains unclear how DNMTs coordinate to achieve genome stability. Furthermore, understanding the mechanisms that control the reprogramming of IAP methylation states will lead to a better understanding of transgenerational epigenetic inheritance and the fetal origins of adult disease [21–23]. For example, environmental exposure-induced increases in methylation of an IAP upstream of the *Agouti* gene in early embryos explains the variation of coat color and propensity for disease in adult mice [24, 25].

It has been demonstrated that DNA methylation has a strong correlation with repression of gene transcription by cellular transfection of unmethylated and methylated counterparts of the same sequence in vitro [26] and using transgenic mice in vivo [27]. In line with this, DNMT1 inhibitor drugs have been developed to relieve the inhibition of DNA methylation on silenced tumor suppressor genes. However, progress with these inhibitor drugs in the treatment of various cancers has lagged behind expectations. While the US Food and Drug Administration has approved two DNMT1 inhibitor drugs for the treatment of myelodysplastic syndromes, many more drugs have failed in clinical trials [28]. A recent report that DNMT1 inhibitor drug-induced demethylation causes the re-activation of both tumor suppressor genes and pro-metastatic genes raises concerns about promoting metastasis [29]. Such reports reveal the complexity of the underlying mechanisms. A better understanding of the mechanisms of re-activation of silenced tumor suppressor genes would help in the development of next-generation DNMT inhibitor drugs.

Recently, methylation of transcribed regions has been proposed to promote gene expression [30]. RNA-seq studies in wild-type (WT) and DNMT1^−/−^/3a^−/−^/3b^−/−^ triple knockout (TKO) embryonic stem (ES) cells, however, indicated that the expression of the majority of genes in the mouse genome did not change much [31]. Because of the previous lack of methylome data, the underlying mechanisms for how DNA methylation regulates gene expression have not been characterized [31].

By analyzing deep sequencing-generated base-resolution methylomes of WT and a series of DNMT knockout (KO) mouse ES cells [32–34], we identified previously unappreciated roles for DNMTs in the establishment and maintenance of DNA methylation patterns. We present a refined model that is better suited to explain how distinct DNMT enzymatic activities maintain strand-balanced methylation, suppress TEs, and regulate a subset of genes that have high methylation and low CG density at promoter regions.

## Results

### Base-resolution methylomes of DNMT knockout ES cells

To understand the establishment and maintenance of DNA methylation patterns, we used bisulfite deep sequencing (BS-seq) to generate base-resolution DNA methylomes of WT, DNMT1^−/−^ (1KO), DNMT3a^−/−^/3b^−/−^ (DKO), and DNMT1^−/−^/3a^−/−^/3b^−/−^ (TKO) cells [32–34]. To rule out the scenario where DNMTs retain enzymatic activity from alternative splicing in KO cells, as observed in the disruption of human DNMT1 [35], we performed RNA-seq analyses to detect any potential alternative splicing (Figure S1 in Additional file 1). Interestingly, while alternative splicing of DNMT1 was observed in both 1KO and TKO cells, the enzymatic domain encoded by exons 32–33 were disrupted. The presence of alternative splicing in both human and mouse DNMT1 KO cell lines suggests DNMT1 may play roles that are independent of its methylation activity. Similarly, DNMT3b seemed to have weak alternative splicing, but enzymatic domains of both DNMT3a and DNMT3b were depleted in either DKO or TKO cells (Figure S1 in Additional file 1). We next examined the DNA methylomes of these cell lines at base resolution with BS-seq. Genomic DNA from different cell lines were treated with bisulfite and sequenced with the Illumina HiSeq platform. The sequencing data are of high quality (Figure S2 in Additional file 1) and the sequencing depth is comparable to that of recent mouse DNA methylomes [36] (Figure S3 in Additional file 1). To improve the alignment of sequenced reads to repetitive elements to gain insights into the effects of DNMT on genome stability, we developed and used a new alignment algorithm, *a*nchored *c*luster *e*nd-mapping (ACE-mapping), which has comparable performance to published methods (see detail in Materials and methods).

### CG density predetermines the magnitude of methylation reduction

**Fig. 1:**
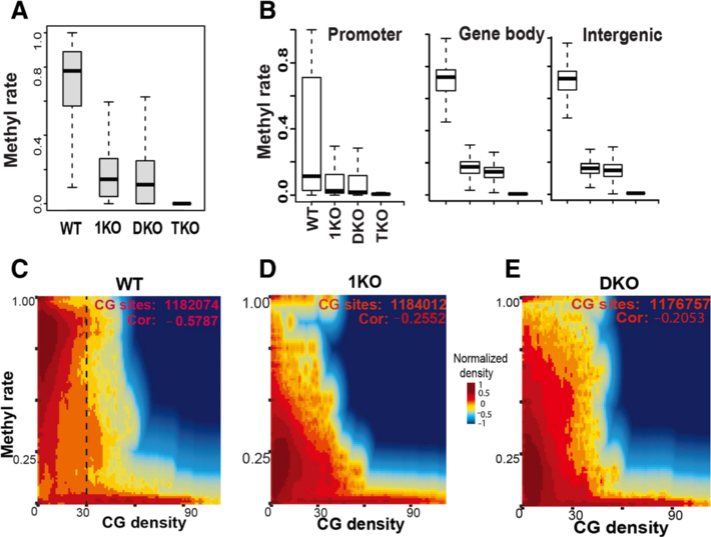
CG density predetermines the magnitude of methylation reduction. **a** Methylation reduction in DNMT KO ES cells. Methylation rates were calculated as described in materials and methods and all CG sites with coverage >6 were used. **b** Methylation at promoter, gene body, and intergenic regions in the mouse genome. **c**–**e** Correlation of DNA methylation with CG density. A two-dimensional density transformation based on kernel density estimates (KDE) was applied to reflect the relative number of CG sites that share similar methylation levels and CG density. The colors from dark blue to light blue, yellow, light red, and dark red, represent the relative number of CG sites, i.e., KDE density. The numbers of CG sites with enough coverage and Pearson correlation coefficients are shown. CG density is defined as the number of CG sites within a 600-bp window. Data in (**c**–**e**) are from chromosome 3 only

With base-resolution methylomes of WT and DNMT-deficient cells, we aimed to investigate the mechanisms of how DNA methylation patterns are established and maintained. Loss of DNMT function leads to dramatic decreases in methylation levels in 1KO, DKO, and TKO cells (Fig. 1a, b; Figure S4 in Additional file 1). The median methylation level in WT is around 78 %, which drops to below 20 % in 1KO and DKO cells, and close to 0 % in TKO cells (Fig. 1a). These observations are consistent with previous studies demonstrating that the TKO cells have ~0.4 % methylation compared with WT by randomly sequencing bisulfite-treated genomic DNA [33]. In 1KO and DKO cells, 42 % and 56 % of CG sites, respectively, have methylation levels <10 % compared with WT, and only very few sites have methylation levels >50 % (Figure S4b–e in Additional file 1). Methylation loss due to lack of maintenance DNMT1 is different from that caused by disruption of de novo DNMT3a/3b. As indicated in Figure S5 in Additional file 1, although most CG sites have low methylation in both 1KO and DKO cells, there are sites that remain highly methylated in each cell type. No significant differences were observed in methylation reduction at promoters, gene bodies, or intergenic regions (Fig. 1b).

Next, we focused on the role of CG islands in shaping the landscape of methylation patterns. The distribution of CG sites is one of the major factors shaping DNA methylation patterns [9, 36]. We used CG density, defined as the number of CG sites within a window of 300 bp upstream and downstream of the CG of interest (see Materials and methods), throughout our studies. As expected, we observed a negative correlation between CG density and methylation (Fig. 1c–e). Unexpectedly, our analyses revealed that a CG density of ~30 is a threshold that predetermines the methylation level in WT cells and the magnitude of methylation reduction in DKO and 1KO cells (Fig. 1c**–**e). The high methylation rates of sites with CG density <30 are significantly reduced in 1KO and DKO cells, whereas methylation of sites with density >30 was essentially unchanged in these cells.

We subsequently examined how methylation is affected by DNMT deficiency in non-CG contexts, including GC, CWG, and GWC (W = A or T). These contexts have very low methylation levels compared with CG (Figure S4a in Additional file 1) but lack of DNMT enzymes led to methylation decreases in these sequences as well. The trend is similar between GC and GWC, but the CWG context is special, having extremely low methylation in all four cell lines (Figure S4a in Additional file 1).

### De novo DNMT3a/3b and maintenance DNMT1 work complementarily and simultaneously to retain methylation patterns

The faithful copying of cytosine methylation by DNMT1 from parental to daughter strand has been proposed to ensure inheritance of epigenetic information across generations; however, CHH (H = A, T, or C) methylation cannot be maintained in such a way because the daughter strands have no cytosines in the triplet. CHH methylation therefore suggests a more complex model. We propose that there are at least two scenarios to potentially explain epigenetic inheritance: 1) DNMT1 is responsible for maintenance of symmetric CG methylation, but DNMT3a/3b are designated to methylate CHH sites during each cycle of DNA replication; or 2) all three DNA methyltransferases are needed to maintain CG methylation symmetry as well as to transmit CHH methylation across generations.

**Fig. 2:**
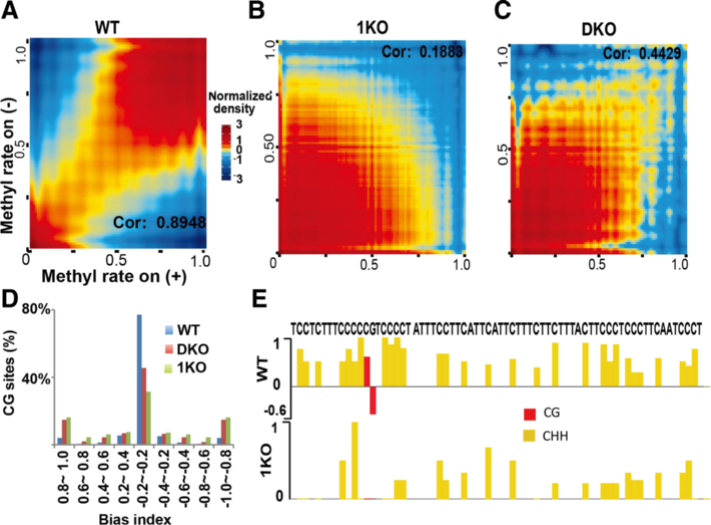
Both DNMT1 maintenance-dependent and -independent activities are required for attaining full methylation. **a**-**c** Correlation of CG methylation on the sense and antisense DNA strands in WT, 1KO, and DKO cells. The CG sites with coverage ≥10 on each strand were used. A two-dimensional transformation was applied and color represents two-dimensional transformated density. *Cor* correlation coefficient. **d** Distribution of bias index (see Materials and methods for details). **e** CHH methylation in 1KO cells unveils the existence of DNMT1 maintenance-independent patterns

To investigate the plausibility of these scenarios, we analyzed CHH methylation and asymmetric CG methylation in our data. As expected, the symmetry of CG methylation in WT cells is high, with a Pearson correlation coefficient (PCC) of 0.8948 (Fig. 2a). Without DNMT1 activity, the PCC in 1KO cells is 0.1883. While the correlation is low, there are still many CG sites with symmetric methylation (Fig. 2b). The symmetry of these CG sites in the absence of DNMT1 maintenance suggests that DNMT3a/3b continually add methyl groups to cytosine on daughter strands after each cycle of DNA replication, consistent with DNMT3a/3b being able to directly interact with Np95 [37]. In comparison, DKO cells have a PCC of 0.4429 (Fig. 2c), indicating that DNMT3a/3b de novo activities are required to maintain CG methylation symmetry to the level of WT cells. Next, we measured the level of methylation bias at each CG site on both the sense and antisense strands by introducing the concept of a bias index (BI), defined as the difference between CG methylation rates on the sense and antisense strands and normalized by the sum of rates on both strands. About 80 % of CG sites have a BI in the range of −0.2 to 0.2 in WT cells. In contrast, only 30 % of sites in 1KO cells and 40 % of sites in DKO cells are within this range (Fig. 2d). Both methylation correlation and BI analyses were reproducible in two independently prepared replicates in three cell types (Figures S6 and S7 in Additional file 1). These data further indicate that DNMT1 alone, without DNMT3a/3b, cannot maintain genome-wide symmetric CG methylation in 1KO cells and that DNMT3a/3b in the absence of DNMT1 can provide some degree of methylation symmetry. These mechanistic insights are consistent with the fact that several species of marine algae have no *DNMT1* gene, but still show symmetric methylation [38].

While the results discussed above (Fig. 2b–d) apply to CG methylation, we were also interested in CHH methylation. Figure 2e shows one selected region from chromosome 3 with methylation at 29 CHH sites and one CG site in WT and 1KO cells. The existence of CHH methylation in the absence of DNMT1 activity in 1KO cells validates our expectation that CHH methylation activities arise either from de novo DNMT3a/3b or from RNA-specific DNMT2 (only DNMT1/2/3a/3b have enzymatic activity). Such CHH methylation is integral to the final methylome (Fig. 2e), and further reinforces our understanding that DNMT1 maintenance-dependent and -independent methylation activities have some kind of 'division of labor'.

### Repression of LTRs is mainly mediated by DNMT1 activity

With only DNMT1 activity, DKO cells have significant methylation reduction globally (Fig. 1a, b). However, some CG sites in DKO cells have methylation levels similar to WT (Figure S8 in Additional file 1). In other words, DNA methylation levels of these CG sites are not significantly reduced upon the loss of DNMT3a/3b de novo methylation activity. To find out whether such sites are sporadically scattered across the genome or cluster together to form a region or domain, we introduced the deletion index to measure the level of reduction of DNMT deficiency-induced methylation. Most CG sites have large deletion indexes (Figure S9a, b in Additional file 1), and the deletion index is larger at CG sites with higher methylation in the WT (Figure S9c, d in Additional file 1). Similar to Fig. 1b, there are no substantial differences among the deletion indexes of promoter, gene body, exon, intron, or intergenic regions (Figure S9e, f in Additional file 1).

**Fig. 3:**
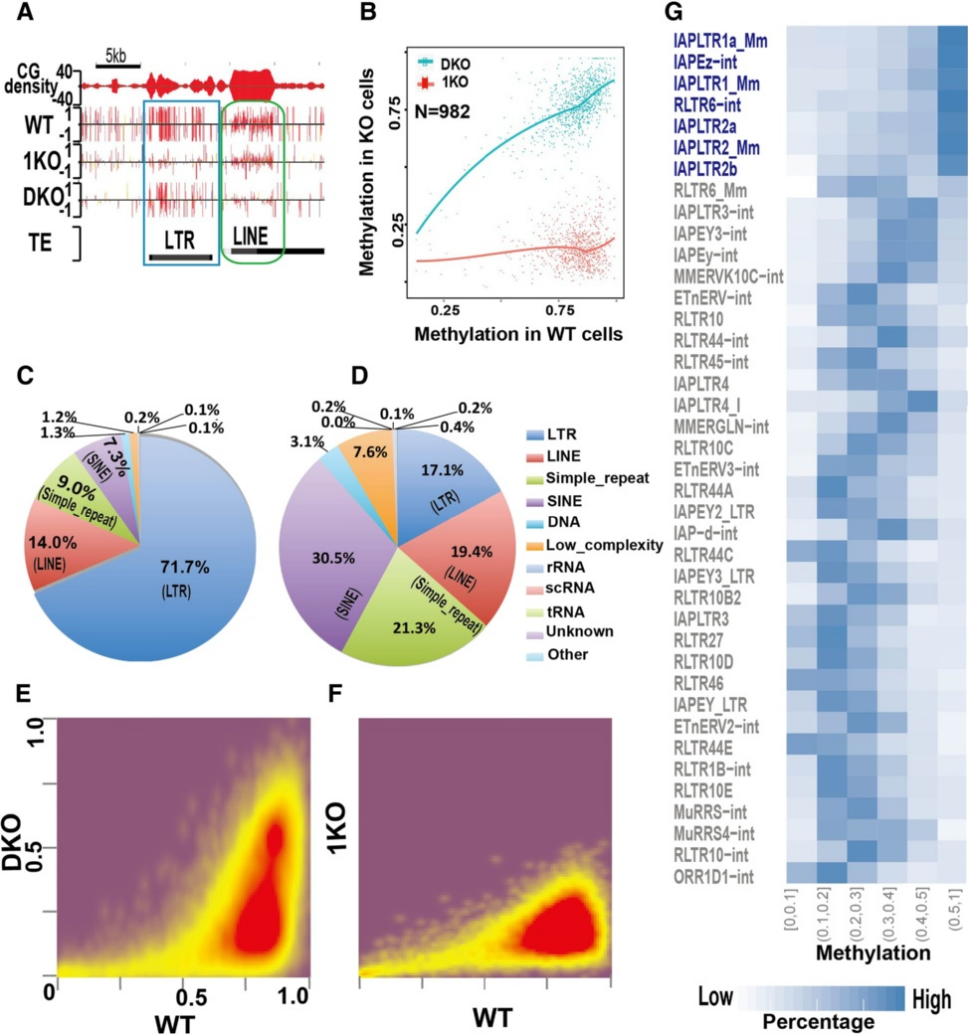
DNMT1 maintenance-dependent methylation patterns are critical for suppressing LTR retrotransposons. **a** Methylation at two neighboring TEs, LTRs (*blue rectangle*) and LINEs (*green oval*), in DKO and 1KO cells. **b** Methylation of RRMRs in WT and KO cells. Each dot represents the overall methylation of one RRMR. **c** Overlap of DKO cell RRMRs with all repetitive elements in the mouse genome. *scRNA* small-scan RNA, *SINE* short interspersed nuclear element. **d** Composition of repetitive elements in the mouse genome. **e**, **f** Correlation of LTR methylation in DKO or 1KO cells with WT. Color represents two-dimensional transformed density. **g** DNMT1 displays different efficiencies during the maintenance of LTR subtype methylation. The percentage of members within each LTR subtype falling into each of six ranges was calculated and transformed to color intensity. LTR subtypes are ordered by percentage within the highest methylation range, 0.5–1, then second highest. Shown in blue are the top 7 LTR subtypes whose members retain high methylation in DKO.

We then identified genomic regions (defined as reduction-resistant methylation regions (RRMRs)) bearing more than seven consecutive CG sites, with each site having a deletion index <0.3 in DKO cells. Scanning of the whole genome identified 982 RRMRs (Additional file 2). These regions retain high methylation (around 0.78) in DKO cells while displaying significantly decreased methylation in 1KO cells (Fig. 3a, b). The level of methylation in 1KO cells (around 0.18) is as low as in other, non-RRMRs of the genome (Fig. 1a). Visual examination of data on the UCSC Genome Browser suggests DKO cell RRMRs are mainly associated with LTRs, one type of TE (Fig. 3a; Figure S10 in Additional file 1). This observation was confirmed by genome-wide analysis: over 98 % of RRMRs in DKO cells overlap with repetitive elements (Figure S11a in Additional file 1), among which 71.7 % are located proximal to or within LTRs (Fig. 3c). This is a significant enrichment (*p* value <0.01; Fisher exact test) considering the percentage of LTRs in all repetitive elements of the mouse genome is only 17.1 % (Fig. 3d). As a comparison, another type of major retrotransposon, LINEs, has only moderate overlap with RRMRs (14 %), similar to the overall LINE percentage in the mouse genome (Fig. 3c, d). Among LTRs, different subfamilies have different preferences for maintaining high methylation in DKO cells, with 80.7 % of RRMRs being located within ERVK family members, much higher than the ERVK family percentage (28.8 %) in the whole mouse genome (Figure S11b in Additional file 1). Each LTR subfamily is further divided into tens or hundreds of subtypes. Similarly, those subtypes exhibit highly variable potential to maintain high methylation in DKO cells (Figure S11c in Additional file 1). Among the top 12 RRMR-overlapping subtypes, eight are IAP-related (Figure S11c in Additional file 1), indicating that DNMT1-mediated methylation is critical for suppressing IAP transcription. This finding provides a molecular explanation for the previous Northern blotting results indicating that DNMT1 knockout leads to dramatically increased IAP transcription in the mouse embryo [20].

The mouse genome contains 851,246 LTRs, comprising around 10 % of the whole genome [39]. To gain a whole picture of methylation of all LTRs in DKO cells, we plotted LTR methylation in 1KO, DKO, and TKO cells against WT after simple filtering to remove the LTRs that are too short or have too few CG sites (Fig. 3e, f). The LTRs in 1KO cells all have similar methylation levels (Fig. 3f). In contrast, methylation patterns of LTRs in DKO cells seem to form a ‘spindle’ shape. The bottom part of the spindle has a methylation level of around 18 %, and the top has a methylation level of over 50 % (Fig. 3e); in WT, these two parts have similar methylation levels (Fig. 3e). As expected, extremely low methylation was found within LTRs in TKO cells (Figure S12 in Additional file 1). The separation into high and low methylation in DKO cells is independent of sequencing coverage (Figure S13 in Additional file 1).

As revealed by the analyses of the overlap between RRMRs and LTR families, LTR families exhibit different potential to retain high methylation, with ERVK having the highest potential and ERV1 having the second highest (Figures S11b and S14a, b in Additional file 1). Next, we drilled down to ask whether LTR subtypes have different potential to retain high methylation in DKO cells. As shown in Fig. 3g and Figure S15 in Additional file 1, such differences exist. Most members in the top seven subtypes have methylation levels between 50 % and 100 %, while members in the other LTR subtypes mainly have lower methylation levels ranging between 0 % and 50 % (Fig. 3g; Figures S15 and S10 in Additional file 1). These subtype-specific methylation differences within LTRs are specifically related to DNMT1 because such differences do not occur in 1KO and WT cells (Figure S14c in Additional file 1). Collectively, we conclude that DNMT1-dependent methylation activity is essential to maintain methylation and suppression of retrotransposon LTRs in the mouse genome.

### Repression of LINEs is mainly mediated by DNMT3a/3b activity

**Fig. 4:**
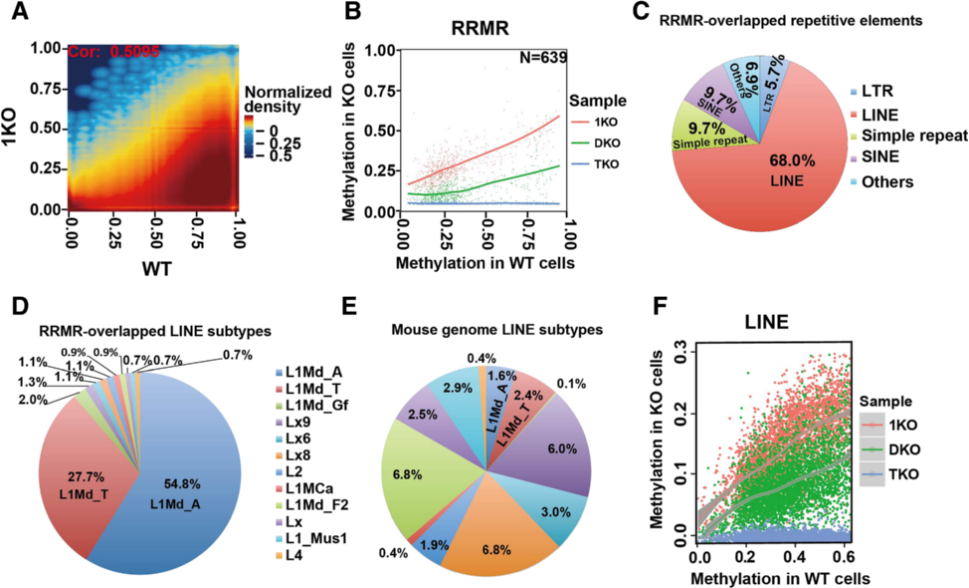
DNMT3a/3b-dependent (i.e., DNMT1 maintenance-independent) methylation patterns are critical for suppressing LINE retrotransposons. **a** Correlation of methylation in 1KO and WT cells. CG sites with coverage ≥10 in both cells were used. Color represents two-dimensional transformed density. **b** Methylation of RRMRs in three different ES KO cell lines relative to WT. **c** Overlap of 1KO cell RRMRs with repetitive elements in the mouse genome. *SINE* short interspersed nuclear element. **d** Overlap of 1KO cell RRMRs with LINE subtypes. Shown are percentages of each LINE subtype out of all LINE subtypes overlapping with RRMRs. **e** Percentages of different LINE subtypes in the mouse genome. **f** Methylation of LINEs in three different ES KO cell lines relative to WT

Similar to DKO cells, the methylation of some CG sites in 1KO cells is relatively well maintained compared with a globally significant reduction (Figs. 3a and 4a). In other words, DNA methylation levels of these CG sites are not significantly reduced upon the loss of DNMT1 activity. Similar to analyses in Fig. 3b, we identified RRMRs (n = 639) exhibiting higher methylation in 1KO than in DKO and TKO cells (Fig. 4b; Additional file 3). The majority of these RRMRs are located within or close to repetitive elements (Figure S16a in Additional file 1). Visual inspection on the UCSC Genome Browser indicated that these RRMRs overlap LINEs (Figs. 3a and 4c; Figure S17 in Additional file 1). Whole genome analysis indicated that 68 % of RRMRs fall within LINEs, much higher than the LINE percentage (19.4 %) in the mouse genome. In contrast, only 5.7 % are within LTRs, which is lower than the LTR percentage in the mouse genome (17.1 %) (Figs. 3d and 4c). Like LTRs, LINEs are composed of hundreds of subtypes. These subtypes apparently have different capabilities to retain high methylation in 1KO cells, with 54.8 % and 27.7 % of RRMRs overlapping L1Md_A and L1Md_T subtypes, respectively, far beyond their percentage in all LINE subtypes (Fig. 4d, e; Figure S16b, c in Additional file 1). Interestingly, the overlapping of LINEs with RRMRs is confined to the promoter region of LINEs (Fig. 3a; Figure S17 in Additional file 1), suggesting a critical role of such high methylation in repressing LINE retrotransposition and/or transcription. Next, we analyzed all LINE promoters in the mouse genome after simple filtering to remove short and low CG density LINEs. As with the findings using the RRMR strategy, these results suggest DNMT3a/3b is crucial for maintaining methylation in LINE promoters. The methylation of LINEs in 1KO cells is always higher than that in DKO cells (Fig. 4f).

The above analyses indicate that: 1) LTRs exhibit special methylation retention from DNMT1 activity after DNMT3a/3b knock-out (Fig. 3); 2) LINE promoters exhibit similar retention from DNMT3a/3b activities after DNMT1 knock-out (Fig. 4); and 3) protein-coding genes do not show this methylation retention (Fig. 1b; additional examples in Figures S10 and S17 in Additional file 1). Therefore, our results provide a molecular explanation of previous Southern blotting results [33, 34] (see summarized data from previous reports in Figure S18 in Additional file 1). To confirm this variability, methylation along the body of these three elements was further analyzed. Genes, LINEs and LTRs in the mouse genome were divided into 10 groups according to their CG density. Their bodies were divided into 50, 20, and 15 bins, respectively, with each bin having the same number of CG sites. An 'overall methylation rate' and median CG density were calculated for each bin within each group (Figure S19 in Additional file 1). As expected, LTRs show higher methylation in DKO than 1KO cells and this high methylation spreads through the whole LTR body, while LINEs show higher methylation in 1KO than DKO cells and this high methylation is restricted to promoter regions (Figure S19 in Additional file 1). Only the LINEs or LTRs with high CG density display such differences, while those with low CG density retained similar levels of methylation between 1KO and DKO cells.

Strikingly, the role of CG density in shaping methylation patterns is different for the three elements. For genes and LINEs, the CG density is inversely correlated with methylation level. While for LTRs, this trend seems to be reversed, with higher CG density being accompanied by higher methylation (compare CG density and WT methylation in Figure S19 in Additional file 1). It is clear that LINE promoters always have higher methylation than gene promoters (compare gene and LINE methylation in WT, 1KO, and DKO cells in Figure S19 in Additional file 1). This difference does not result from the fact that CG density in gene promoters is higher than that in LINE promoters, because a select set of genes with similar CG densities as LINE promoter regions also exhibit lower methylation (Figure S20 in Additional file 1). Intriguingly, LINEs in 1KO cells do not display the typical pattern of methylation — slowly increasing throughout the gene body — but display similar methylation across the whole LINE body. This observation indicates that DNMT3a/3b in DNMT1^−/−^ 1KO cells have equal chances to methylate CG sites regardless of CG density. However, this typical pattern is apparent in WT cells, whose only difference with 1KO cells is the intact DNMT1, suggesting that DNMT1 is responsible for this pattern by preferentially retaining methylation at regions of low CG density. This idea is further confirmed by the observation that the DKO cells, which have lost DNMT3a/3b activities but retain intact DNMT1, still have the typical methylation pattern. It is noticeable that the above observations are independent of coverage depth. As shown in Figure S21 in Additional file 1, the three groups with different coverage depth, GR1 (lowest coverage), GR2 (medium), and GR3 (highest), have very similar methylation patterns in WT, 1KO, and DKO cells for both LINEs and LTRs.

### Hypomethylation induces or unexpectedly represses the transcription of protein-coding genes with low CG density

**Fig. 5:**
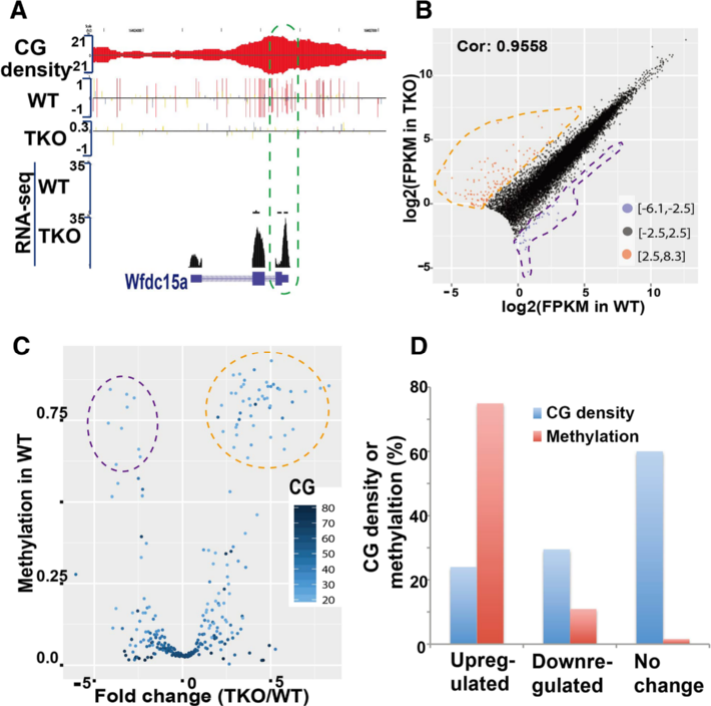
Global hypomethylation leads to expression changes of genes with low CG density at promoter regions. **a** *Wfdc15a*, with low CG density, was induced in TKO cells. **b** Correlation of gene expression in WT and TKO cells. Genes (n = 13,436) with FPKM >0 in both samples and FPKM sum of two samples >1 were included in the analysis. Up- and down-regulated genes with log2-transformed fold change >2.5 or < −2.5 are shown as *red* or *blue dots*, respectively. **c** Genes with high methylation and low CG density in promoters display significant changes in gene expression. The genes shown in panel (b) were cut into 500 equal-length subgroups according to expression fold change between WT and TKO cells. The median fold change in each subgroup was plotted against overall methylation in the same subgroup. CG number in each subgroup is shown by blue color intensity. **d** CG density and methylation rate (in WT cells) of genes with up-regulated, down-regulated, or unchanged expression around the TSS (500 bp up- and downstream)

Our data from extensive bioinformatic analyses indicate that DNMT1-dependent and -independent methylation patterns are responsible for inhibiting the transcription of retrotransposon LTRs and LINEs, respectively. Next, we investigated how reduced methylation can affect transcription of protein-coding genes. Understanding these mechanisms might help to improve the application of DNMT1 inhibitors in the induction of silenced tumor suppressor genes during treatment of various cancers. First, we examined methylation at the promoters and transcription start sites (TSSs) of genes in four ES cell lines (Figure S22 in Additional file 1). In WT cells, DNA methylation around TSSs is negatively correlated with gene transcription, as expected. The trend is conserved in 1KO and DKO cells, even though their methylation levels are significantly reduced (Figure S22 in Additional file 1). Due to the hypomethylation in TKO cells, such negative correlation cannot be detected (Figure S22 in Additional file 1). Except for a minority of genes, however, genes in TKO cells have similar expression levels as in WT cells (Fragments Per Kilobase of exon per Million fragments mapped (FPKM) PCC = 0.9558) (Fig. 5b), consistent with a former report [31]. On the other hand, some genes do exhibit changes of expression in TKO cells (Fig. 5a, b; Additional file 4). These genes show special features in terms of DNA methylation and CG density (Fig. 5c, d; Figures S23 and S24 in Additional file 1). The average promoter methylation reaches 75 % for up-regulated genes (fold change >2.5), drops to 11 % for down-regulated genes (fold change < −2.5), and further drops to 2 % for unchanged genes. Meanwhile, we observed that up-regulated, down-regulated, and unchanged genes have CG densities of 24, 30, and 60, respectively (Fig. 5d).

To more thoroughly examine these special features, genes were divided into 500 subgroups according to fold change between TKO and WT cells. The median fold change in each subgroup was plotted against overall promoter methylation of WT cells in the same subgroup. Genes with fold change around 0 have low DNA methylation and high CG density, whereas genes with large fold changes (up-regulated) show significantly higher methylation and lower CG density (Fig. 5c). Unexpectedly, a small group of down-regulated genes also displayed hypermethylation. While the up-regulation of genes due to the loss of hypermethylation is in line with current understanding of the inhibitory role of promoter methylation, the down-regulation of genes upon the loss of promoter hypermethylation is unexpected. Whether it is an indirect effect, such as the expression of a transcription repressor, remains to be determined. At this resolution, gene body methylation seems to have no effect on expression deregulation in TKO cells (Figure S25 in Additional file 1).

## Discussion

**Fig. 6:**
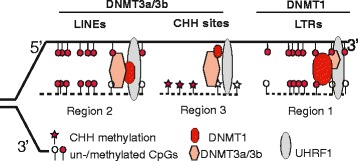
A revised model illustrating complementary DNMT3a/3b de novo activity and DNMT1 maintenance activity at three genomic regions. Via interacting with proteins such as UHRF1 at replication forks, DNMT1 plays a dominant role in maintaining methylation at retrotransposon LTRs (region 1), whereas DNMT3a/3b are mainly responsible for methylation at retrotransposon LINEs (region 2) or genomic regions with CHH sites (region 3). Loss of DNMT3a/3b or DNMT1 results in hypomethylated retrotransposon LTRs or LINEs

Our base-resolution data from BS-seq reveal a clear picture that all three DNA methyltransferases, DNMT1 and DNMT3a/3b, are required at the stages of both establishment and maintenance in the mouse genome, consistent with DNMT1-independent symmetric methylation reported previously in marine algae [38]. Specifically, we found that DNMT3a/3b de novo activities complement the inefficiency of DNMT1 in achieving symmetric CG methylation (Figs. 2 and 6). Consistently, hairpin-bisulfite sequencing of DNMT KO ES cells at limited regions, including LINEs and a few protein-coding genes, demonstrates that DNMT3a/3b contribute to the symmetry of CG methylation [11]. In line with this notion, DNMT3a/3b are inefficient at LTRs, whereas DNMT1 is efficient (Figs. 3 and 4). Similarly, DNMT1 is inefficient at LINEs, whereas DNMT3a/3b are efficient. These data prompted us to present a new model to illustrate the ‘division of labor’ between DNMT3a/3b activities and DNMT1 activity in the maintenance and establishment of methylation patterns, including CG and CHH methylation, at genomic regions with unique features (Fig. 6). Our model is consistent with a former proposal that DNMT3a/3b compartmentalize to repetitive elements to complement the methylation inefficiency of DNMT1 [13, 40].

Retrotransposition causes genome instability and many human diseases [17]. On the other hand, it helps to increase genetic diversity [19]. Our results reveal that DNMT1-dependent and -independent methylation activities have distinct roles in the suppression of retrotransposon LTRs and LINEs, respectively. This may hint at why cells change the expression levels of different types (and variants) of DNMTs during development, with such changes providing cells a chance to control the jumping of retrotransposons in neurons for neuronal diversity or in germ cells for evolutionary benefits [19, 41–44]. Meanwhile, the sufficiency of DNMT1 alone in maintaining IAP methylation (Fig. 3) provides an attractive explanation of why agouti mice can preserve the methylation of the inserted IAP retrotransposons through the female germ line, a phenomenon of ‘epigenetic inheritance’ [45]. It is known that DNMT1 and its short form are expressed in female germ cells in addition to other somatic tissues, whereas expression of DNMT3a/3b is low in somatic tissues. We expect, therefore, that our results help to elucidate transgenerational epigenetic inheritance [23].

With base-resolution methylomes of DNMT KO ES cells, our data suggest that genes with high or low CG density use different mechanisms for maintaining gene silencing. At least, DNA methylation and other epigenetic mechanisms, such as histone modification, seem to play different roles in promoters with high or low CG density. For example, the hypomethylation in TKO cells only re-activates a small number of silenced genes with low CG density. In contrast, many more silenced genes with high CG density are not induced in TKO cells. Therefore, it seems that DNA methylation plays a dominant role in repressing the former, whereas epigenetic mechanisms other than DNA methylation might control the latter. These observations in ES cells are intriguing when compared with a working model of progressive silencing of tumor suppressor genes from early histone methylation-mediated silencing to later DNA methylation-maintained silencing in cancer cells [46, 47] (reviewed in [48]). On the other hand, from both our analyses (Fig. 5) and a previous report [31], the majority of genes in hypomethylated TKO cells (Fig. 1) have similar expression levels as in WT cells. Similar expression of the majority of genes suggests that dense methylation at transcribed regions of active genes (Figs. 1 and 5) may not affect RNA polymerase II elongation, either negatively [49] or positively [30]. Some recent independent investigations are more affirmative [50], whereas others observe a correlation that is not always positive [51]. More investigations are needed, and we are in the process of examining gene expression changes in the restored methylome of TKO cells.

The distinct roles of DNMT1 maintenance-dependent and -independent methylation activities may explain the inefficiency of DNMT1 inhibitor drugs in the treatment of cancers, because only DNMT1-dependent patterns are reduced and DNMT1-independent patterns are not. Combinatorial applications of inhibitors of de novo and maintenance methyltransferases are attractive. Though our insights are from ES cells, we expect that cancer stem cells or somatic cancer cells would have similar behaviors because the overexpression of de novo methyltransferase DNMT3a and/or 3b in different types of cancer has been reported in numerous studies [52, 53]. Lastly, the induction of only genes with lower CG densities indicates the limitation of the application of DNMT1 and maybe future DNMT3a/3b inhibitor drugs.

## Conclusions

We have determined base-resolution DNA methylomes with improved coverage at highly repetitive elements of a series of DNMT knockout ES cells. Our data reveal a complex and complementary coordination between DNMT1 activity and DNMT3a/3b activities for establishing/maintaining methylation patterns, compared with the broadly accepted 'two-step' model. DNMT3a/3b or DNMT1 show(s) inefficiency or preference to maintain/establish methylation patterns at specific genomic regions. Consistently, DNMT1 cannot by itself maintain symmetric CG methylation, whereas de novo methylation activity can establish somewhat symmetric CG methylation in the absence of a maintenance activity (Fig. 2). We conclude that a normal methylome with symmetric CG methylation and CHH methylation really needs the synergistic and complementary actions of DNMT1 and DNMT3a/3b [37] in mammalian genomes.

In line with specific roles of DNMT1 and DNMT3a/3b in the maintenance/establishment of methylation patterns, we demonstrate that DNMT1-dependent and DNMT3a/3b-dependent patterns have a ‘division of labor’ in suppressing retrotransposon LTRs and LINEs, respectively. These novel insights open new avenues to understand biological processes, including transgenerational epigenetic inheritance. For example, different methylation levels of TEs can affect nearby gene expression and thereby phenotype, such as in the agouti mouse. Intriguingly, only genes with low CG density are induced upon hypomethylation in TKO cells, whereas genes with high CG density are not. Together with the separate roles of both DNMT1 and DNMT3a/3b in maintaining/establishing methylation patterns, our results provide potential explanations for the inefficiency of DNMT1 inhibitors alone in the treatment of human diseases.

## Materials and methods

### ES cell culture

Mouse ES cells WT J1, DNMT1 knockout 36c/c (1KO) and DNMT3a/DNMT3b double knock out (DKO) were kindly provided by Drs En Li and Taiping Chen [34, 54]. DNMT1/DNMT3a/DNMT3b triple knockout cells (TKO) were kindly provided by Dr Masaki Okano [33]. Cells were maintained without feeder cells on 0.1 % gelatin coated Petri dish in Dulbecco's modified Eagle's medium (DMEM) supplemented with 15 % fetal bovine serum (ES cell grade), 2 mM glutamine, 10 μM mercapto-ethanol, 100 U/ml LIF, penicillin/streptomycin mixture 100 μg/ml, 1× non-essential amino acids.

### BS-seq library construction

Genomic DNA (1–5 μg) was sheared to 200–500 bp by Covaris S220 sonicator (Covaris, USA). End repair was then performed following the manufacturer’s instructions (End-It DNA end repair kit, Epicentre, USA). After Ampure XP (Sigma, USA) purification, adenine was added to the 3′ end with 3 μl DNA Taq polymerase (M0267S, NEB) and 1 mM dATP in 50 μl reaction solution incubated at 70 °C for 30 minutes. After Ampure XP purification, 1 μl of Illumina Trueseq adaptors were ligated with 4 μl T4 DNA ligase (M0202L, NEB) in 40 μl reaction solution and incubated at 16 °C overnight. Adaptor-ligated DNA fragments of 300–600 bp were collected from 2 % agarose gel, and then bisulfite-treated using an Imprint DNA modification Kit (MOD50-1 KIT, Sigma, USA) according to the manufacturer's instructions. To ensure conversion of uracil to thymine and avoid the potential PCR-induced bias [55], we minimized PCR cycles (6–10 cycles) to amplify the libraries, and fragments of 300–600 bp were then collected using 2 % agarose gel electrophoresis.

### Bisulfite Sanger sequencing

Genomic DNA was bisulfite-treated according to the manufacturer’s instructions (Imprint DNA Modification Kit, Sigma, USA). About 40–50 ng of converted genomic DNA was used for Nested PCR. Primers were designed using Methyl Primer Express 1.0 (Applied Biosystems). Purified PCR products were cloned into pCR2.1 vector (TA Cloning Kit, Invitrogen). White colonies were selected through blue/white screening and sent for Sanger sequencing.

### Anchored cluster end-mapping

We developed the ACE-mapping method to use the feature that both ends of a DNA cluster (a clonal amplification from the same DNA fragment during cluster generation) are sequenced in paired-end sequencing and one uniquely aligned mate pair can be used to rescue its mate when the mate has multiple hits in the genome by using knowledge of the appropriate fragment length in library construction. Similar strategies have been independently used by other mapping methods like MOSAIK [56] and segemehl [57]. ACE-mapping is able to increase the mapping efficiency at highly repetitive elements. The mouse reference genome (UCSC mm9) was subjected to two types of nucleotide replacement by changing C to T (C2T genome) or G to A (G2A genome) and indexes were made using bowtie-build. Reads also underwent nucleotide replacement by changing cytosine in read 1 of paired-end sequencing to thymine and guanine in read 2 to adenine. Mapping was done using bowtie (version 0.12.7) [58] by allowing three mismatches in the leading 40 bases and a Phred score of up to 140 at mismatched positions. Twenty multiple alignments were allowed at most for each read, i.e., using k = 20 when running bowtie.

### Removing PCR amplification biases by monoclonization

PCR of bisulfite-treated DNA sometimes shows favorable amplification of unmethylated sequences, which can cause incorrect representation of real methylation in a sequence-specific and often strand-specific manner [55]. To avoid potential biased amplifications, we included one step of monoclonization in our data processing pipeline by treating all reads derived from the common DNA fragment via PCR as one. The idea of monoclonization is based on the fact that there is a very low chance of chromosomes from different cells being broken at the same positions during sonication. So the chance is very low that both read mates of different DNA fragments align to the same chromosome positions. During data processing, we reduce the multiple read pairs that anchor to the same chromosome positions at both ends to one clone by keeping the read pair that has the highest mapping score. As shown in Figure S3 in Additional file 1, WT cells have 817,717,346 tags (i.e., read pairs), but only 301,635,930 clones. Monoclonization, although decreasing sequencing coverage depth, can alleviate the effects of PCR bias. The number of monoclones could reach 36.9 % to 53.9 % of the total tag number (Figure S3 in Additional file 1). As shown in Figure S26 in Additional file 1, three fragments were generated from three different cells during sonication with two of them methylated and one unmethylated at the indicated CG site. The actual methylation rate (MR) should be 67 %. However, the MR becomes 0.3 on the top strand after PCR because fragments 1 and 2 are amplified 10 times (from 1 molecule to 10) and fragment 3 is amplified 50 times (from 1 to 50) due to PCR bias. After monoclonization, all the 10 or 50 DNA molecules were reduced to one since they map to the same chromosome positions on two ends, which gives a MR of 0.67 again. For the bottom strand, since no PCR bias exists, the MR is always 0.67. Extremely high coverage is required to completely remove PCR bias (unpublished data, Dr. Zhibin Wang at Bloomberg School of Public Health, Johns Hopkins University). Usually, about 30× coverage or less is achieved in current reports (including ENCODE). Under these conditions, monoclonization is able to alleviate, but cannot completely remove, the effects of PCR bias.

### Calculation of methylation levels

We use MR to represent methylation level, which is similar to methylation score used in the study by Stadler et al. [36]. We use the following formula:

$$ \mathrm{S} = \mathrm{r}\mathrm{C}\ /\ \left(\mathrm{r}\mathrm{C} + \mathrm{r}\mathrm{T}\right), $$

where rC is the number of reads that remain C after bisulfite treatment, and rT is the number of reads that become T after bisulfite treatment.

### Calculation of overall methylation

Overall MRs were used to measure the overall methylation of all Cs, CGs, or CWGs (W = A or T) in the whole genome, or in specific elements such as LTRs and LINEs. For overall methylation of a genomic region, we used the following formula:

$$ \mathrm{S} = \Sigma \mathrm{r}\mathrm{C}\ /\ \left(\Sigma \mathrm{r}\mathrm{C} + \Sigma \mathrm{r}\mathrm{T}\right), $$

where Σ is the sum of the rC or rT values on every CG site within the region. Except for the analysis of strand bias methylation, the two cytosines in a CG site were combined together to calculate methylation or overall methylation.

### Bias index

BI was defined to measure the degree of methylation bias on two complementary DNA strands and calculated with the following formula:

$$ \mathrm{BI} = \left(\mathrm{S}\mathrm{n}\ \hbox{-}\ \mathrm{S}\mathrm{p}\right)\ /\ \left(\mathrm{S}\mathrm{n} + \mathrm{S}\mathrm{p}\right), $$

where Sn is the MR on the negative (or antisense) strand, and Sp is the MR on the positive (or sense) strand. CG sites with Sn = 0 and Sp = 0 were considered unbiased (i.e., corrected so that BI = 0, rather than undefined).

### Deletion index and RRMRs

A deletion index (Di) was calculated to measure the degree of methylation deletion/reduction compared with WT that was caused by the knocking-out of DNMTs. It is calculated using the following formula:

$$ \mathrm{D}\mathrm{i} = \left(\mathrm{S}\mathrm{w}\mathrm{t}\ \hbox{-}\ \mathrm{S}\mathrm{k}\mathrm{o}\right)\ /\ \mathrm{S}\mathrm{w}\mathrm{t}, $$

where Swt is the MR in WT, and Sko is the MR in 1KO or DKO cells. RRMRs were searched for considering them as regions having five or seven consecutive CG sites with each CG having a deletion index <0.3 in 1KO and DKO cells, respectively.

### Overlap of RRMRs with repetitive elements

A repetitive element table was downloaded from the UCSC genome browser for mouse genome mm9, which was created using RepeatMasker [59]. Overlap was located using the 'findOverlaps' function in the 'IRanges' package of R software with a gap of 200 bp and 50 bp for 1KO and DKO cell RRMRs, respectively. Visual inspection of the genome browser indicated 1KO cell RRMRs are mainly located at LINE promoter regions. We decided to focus on the promoter regions that are downstream of TSSs and span one-fourth of the interested LINE.

### Analysis of methylation in LINEs and LTRs

We examined the methylation of all LINEs that meet the following criteria: length >1500 bp, CG number >30, and coverage >50 in all four cells. The coverage of a LINE is the sum of coverage at each CG site within that LINE. The analysis was restricted to LINE promoter regions, defined as 100 bp upstream and 1000 bp downstream of the TSS of a LINE. LTRs also underwent similar filtering, and the ones having CG density >9 and coverage >50 in four cells were selected. For LTRs, the full region was used for analysis. The methylation level of a LINE or LTR was calculated using overall methylation method. It should be pointed out that coverage is usually more shallow in repetitive regions of LINEs and LTRs than in a protein coding gene (Figure S21a in Additional file 1). The effect is more obvious in LINEs than LTRs, probably because LINEs are usually longer (could be 6–7 kb), which makes short-read anchoring more difficult. Coverage profiles of LINEs show higher coverage in head and tail marginal regions, but lower in body regions (Figure S21B in Additional file 1). However, the coverage profiles of LTRs do not show such characteristics, probably due to the fact that LTRs are shorter and easier to fully cover using the uniquely anchored mate (Figure S21C in Additional file 1). However, the slight decrease in coverage does not affect the conclusion we made about the roles of DNMT1 and DNTM3a/3b in LINE and LTR regions as shown in Figure S21d, e in Additional file 1.

### Heatmap of methylation in LTR subtypes

LTRs in the mouse genome are classified into 11 families, and further into 471 subtypes or repNames. Each subtype can have as low as 1 or as high as 47,854 members. The subtypes with >50 members after filtering as described in the *Analysis of methylation in LINEs and LTRs* section were used for heatmap generation. All the members under a subtype were divided into six groups according to their methylation level: [0, 0.1], [0.1, 0.2], [0.2, 0.3], [0.3, 0.4], [0.4, 0.5], and [0.5, 1]. Then, the percentage falling into each range was calculated and represented as a color by the geom_tile function in the ggplot2 package of R software.

### Binning of genomic elements

The genomic elements, including genes, LINEs, and LTRs, were divided into bins by two approaches: 1) each bin having the same length or 2) each bin having the same number of CGs. The first method was employed to investigate methylation in gene promoter regions. Gene promoter regions, comprising 4 kb up- and downstream of the TSS, were divided into 40 bins. Genes were sorted first by expression level (FPKM) then by CG number within promoter, and divided into 500 groups, with each group having the same number of genes. Methylation for each bin within each group was calculated by the overall method as described above. The second approach was used to investigate CG density and methylation distribution along genes, LINEs, and LTRs. These three elements were filtered by the criteria of CG density >50, >20, or >15 and length >3000, >1000, >300, respectively. The regions of filtered genes, LINEs, or LTRs were cut into 50, 20, and 15 bins, respectively. The aforementioned genes, LINEs, or LTRs were further divided into ten equal-sized groups based on CG density number. Within each group, bins in the same sequential order among group members were treated together to obtain an overall MR, which was used in Figure S20 in Additional file 1 (bottom three panels). For CG density, the median of all CG sites in the aforementioned bins was used to generate the top panel of Figure S20 in Additional file 1.

### Alignment of RNA-seq data

WT and TKO RNA-seq data were downloaded from the Gene Expression Omnibus (accession numbers GSM727427 and GSM727428) [31]. DKO and 1KO RNA-seq data were generated in this work. STAR (version 2.1.4a, default parameters) was used to align RNA-seq reads to mouse reference genome mm9 (NCBIM37) [60]. Cuffdiff in the Cufflinks package (version 2.1.1) [61] was used to estimate the expression level by FPKM value based on Ensembl annotation database release 67.

### Analysis of the correlation between DNA methylation and gene expression

Fold change was calculated as the log2-transformed ratio between the WT and TKO FPKM value for each gene. Genes were filtered by the following criteria: 1) FPKM value for both WT and TKO >0; 2) sum of FPKM values for WT and TKO >1; and 3) fold change in the range [−6, 6]. Genes passing the filtering (n = 13,436) were cut into 500 equal-length groups by either fold change or WT FPKM. Overall methylation score, median fold change, CG number, and FPKM were calculated for each subgroup. All the CG sites inside the 500 bp up- and downstream of the TSSs within a subgroup were used for overall methylation calculation. Methylation was plotted against fold change using the ggplot2 package in R software with CG density represented as color shades to reveal the impact of promoter DNA methylation and CG number on gene deregulation. FPKM values were log2 transformed and plotted against log2-transformed methylation score to reveal the expression level of deregulated gene subgroups using the same R package with deregulation status as color aesthetics.

### Methylome data

The methylome data in this report have been deposited in the Gene Expression Omnibus (accession number GSE61457).

## Additional files

Additional file 1:
**Supplemental Figures S1–S26.**
Additional file 2:
**Table listing identified RRMRs in DKO cells.**
Additional file 3:
**Table listing identified RRMRs in 1KO cells.**
Additional file 4:
**Table listing gene expression in FPKM of WT and TKO cells.**

## Abbreviations

1KO: DNMT1 knockout
ACE: anchored cluster end
BI: bias index
BS-seq: bisulfite deep sequencing
DKO: DNMT3a/3b double knockout
DNMT: DNA methyltransferase
ES: embryonic stem
IAP: intracisternal A-particle
KO: knockout
LINE: long interspersed nuclear element
LTR: long terminal repeat
MR: methylation rate
PCC: Pearson correlation coefficient
RRMR: reduction-resistant methylated region
TE: transposable element
TKO: DNMT1^−/−^/3a^−/−^/3b^−/−^ triple knockout
TSS: transcription start site
WT: wild type
