# Supplementary material for: Distinct roles of DNMT1-dependent and DNMT1-independent methylation patterns in the genome of mouse embryonic stem cells
Source: Genome Biol. 2015 Jun 2;16(1):115. doi: 10.1186/s13059-015-0685-2 (PMC4474455; doi:10.1186/s13059-015-0685-2)
Supplement: Additional file 1: — Supplemental Figures S1–S26. [file 13059_2015_685_MOESM1_ESM.pdf]

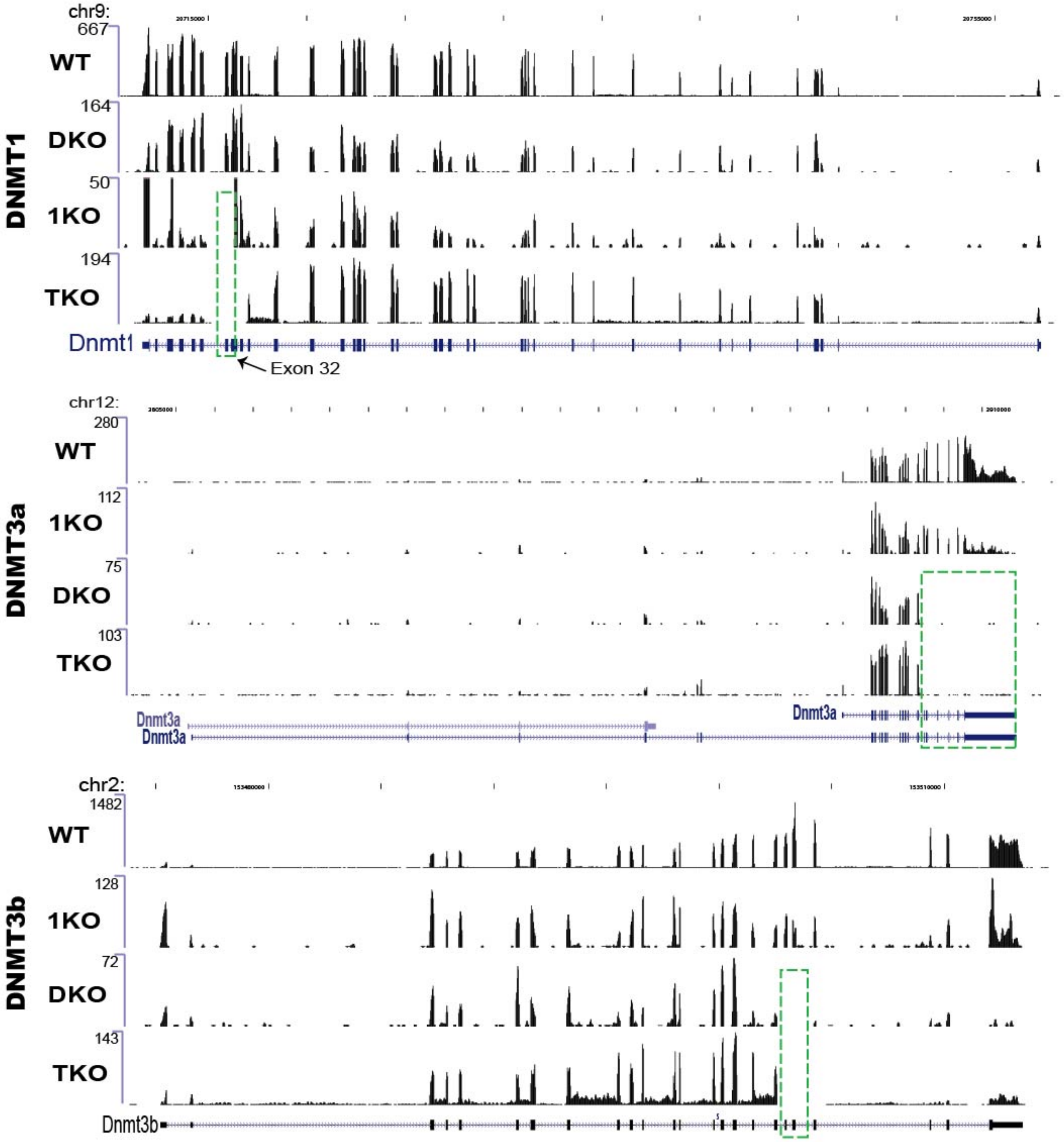

Figure S1. RNA-seq data to show the absence of RNA signals at the enzymatic domain (marked by green rectangles) of DNMT1, DNMT3a, and/or DNMT3b in WT, 1KO, DKO, and TKO cells.

A

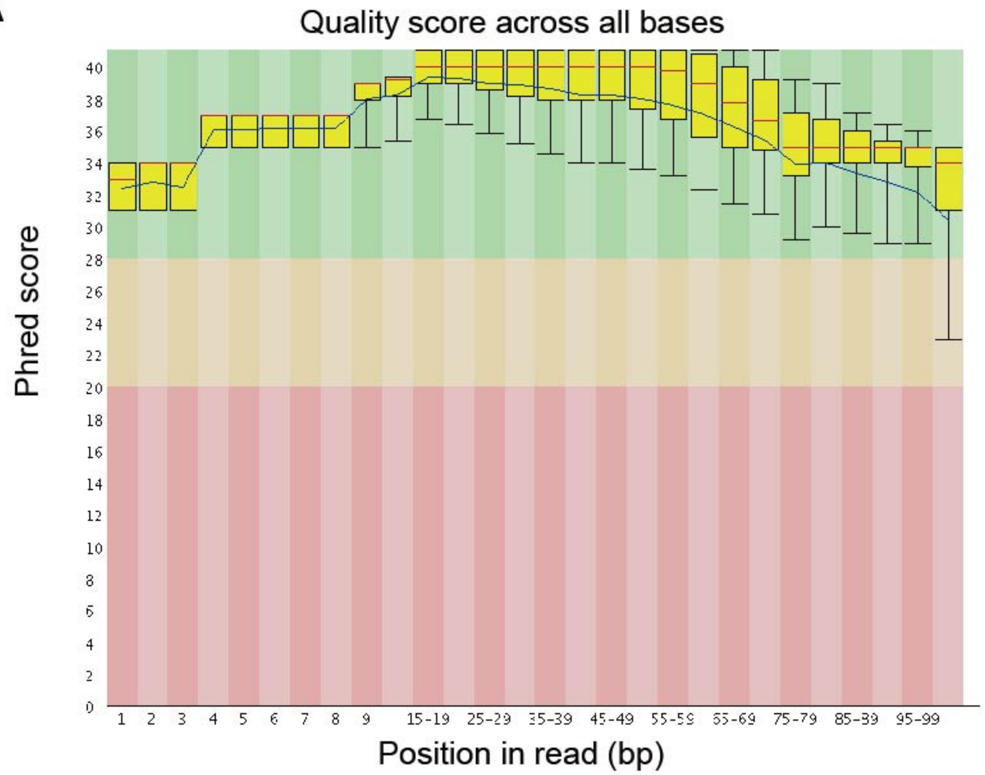

B

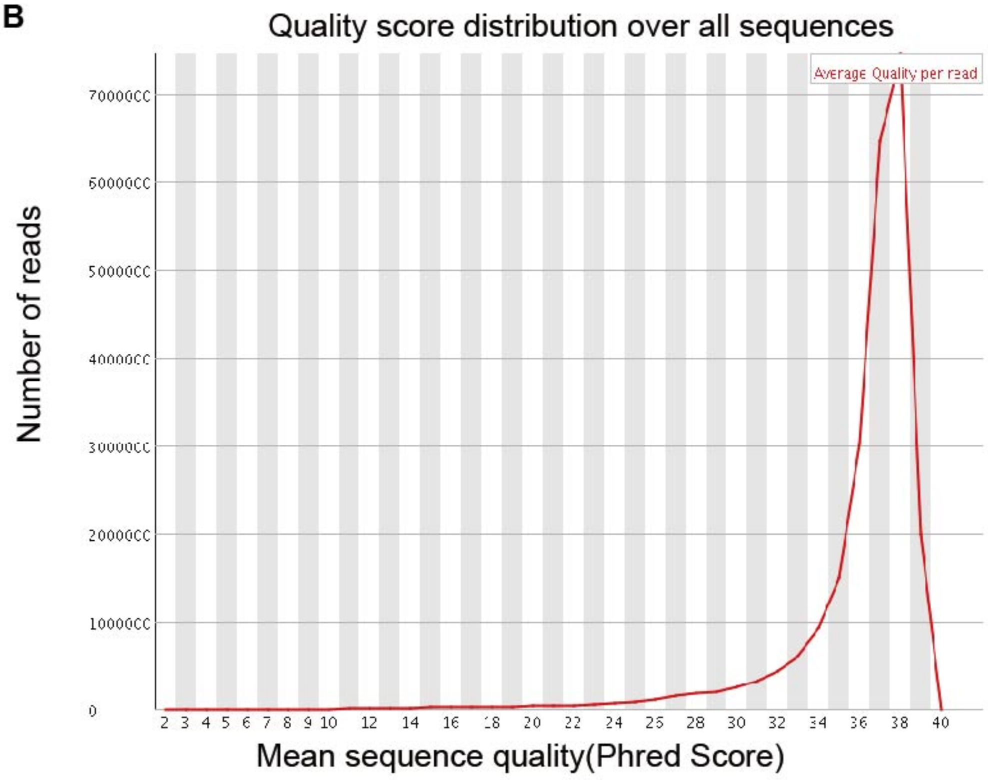

Figure S2. Quality evaluation of sequenced data. (A) High phred score from the 1st to the last base along 100bp read length. (B) Distribution of average phred score of sequenced reads.

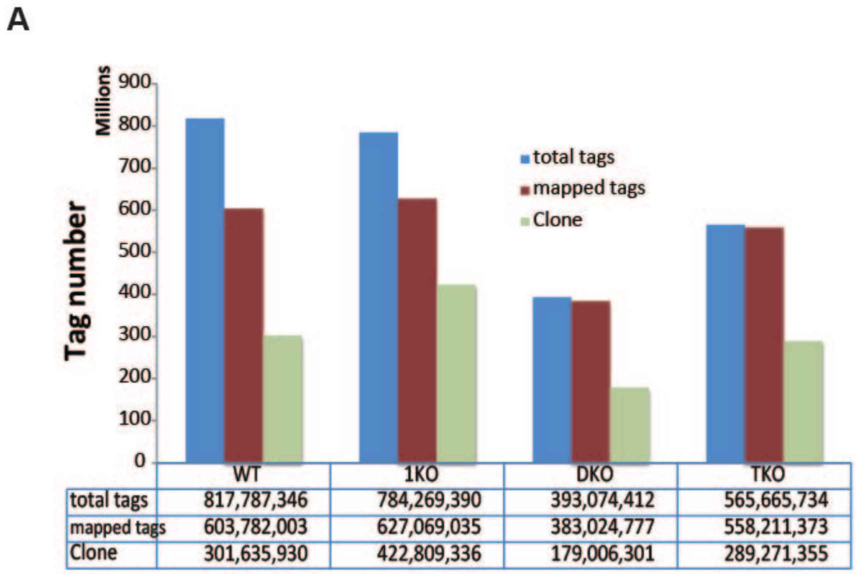

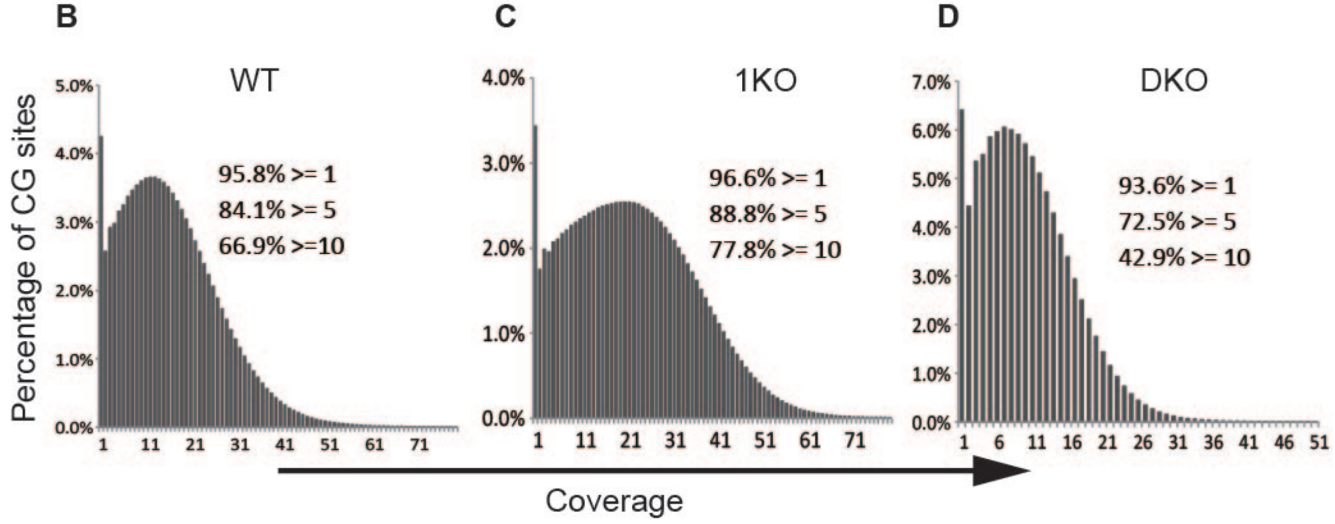

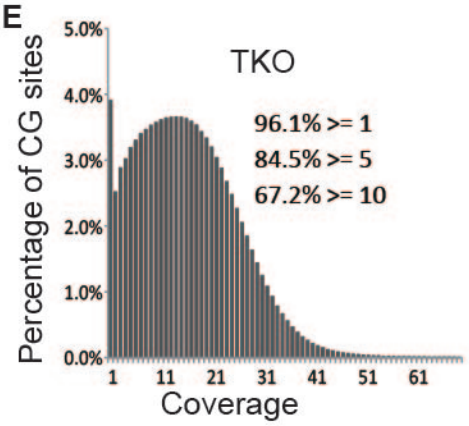

F Coverage of a recent mouse methylome

coverage	≥ 1	≥ 5	≥ 10
percentage of CG	91.9%	84.4%	65.5%

Figure S3. sequencing depth. (A) The number of total tags, mapped tags, and monoclonal tags in four cells. Each monoclonal tag represents one original, unique DNA fragment. (B-E) Coverage of all CG sites in mouse genome in four cells. The coverage is comparable to a previously published mouse methylome study as shown in (F) (*Nature* 2011, 480, 490-495).

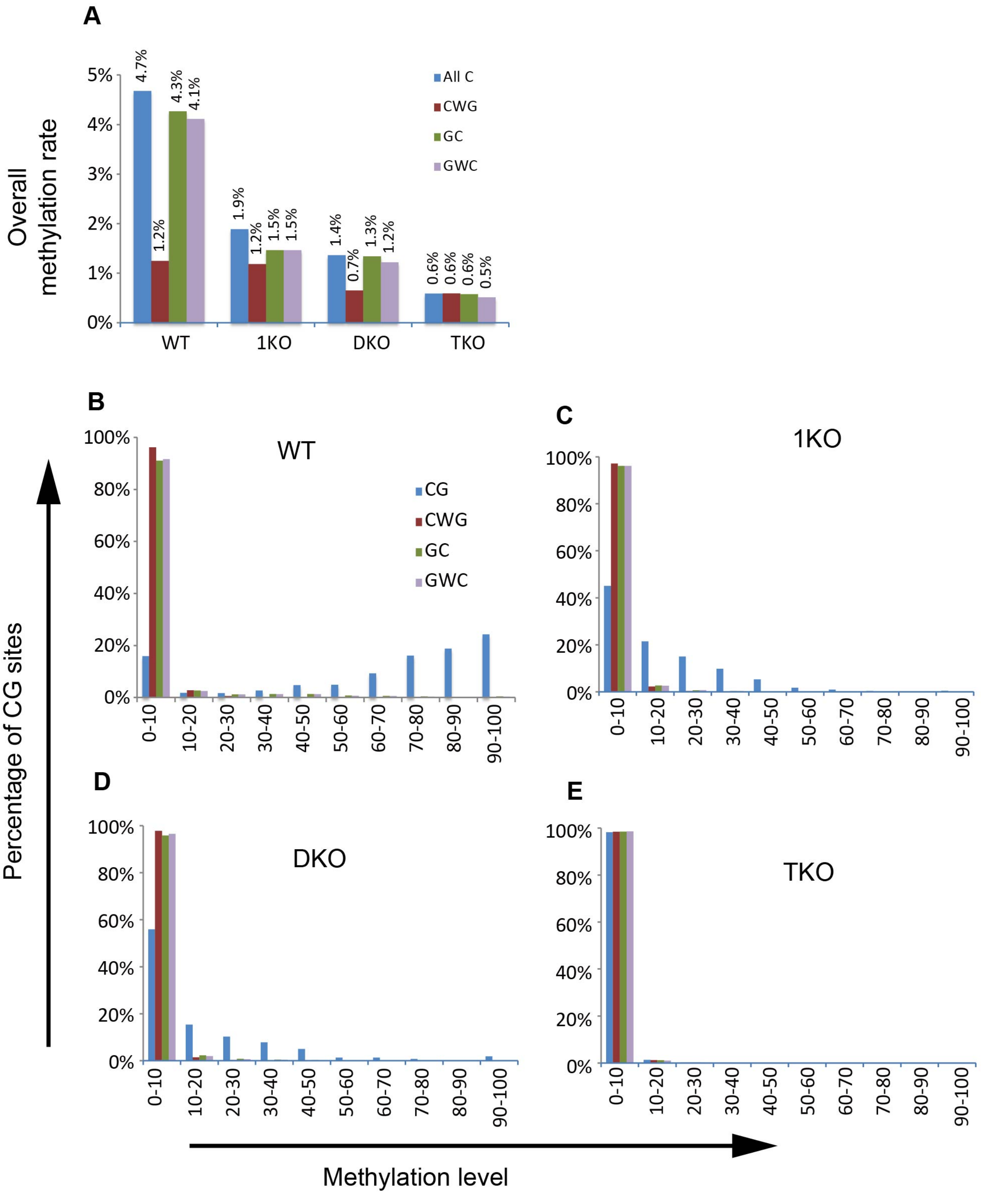

Figure S4. Methylation distribution in four cells. (A) Overall methylation at cytosine context other than CG. All C means all cytosines are considered. The "W" in CWG and GWC refers to A or T. Overall methylation is calculated by treated all cytosines in a specific context as one (see Methods). (B-E) Distribution of methylation at different contexts in four cells. Here, methylation level was independently calculated for each individual site. Overall methylation method was not used.

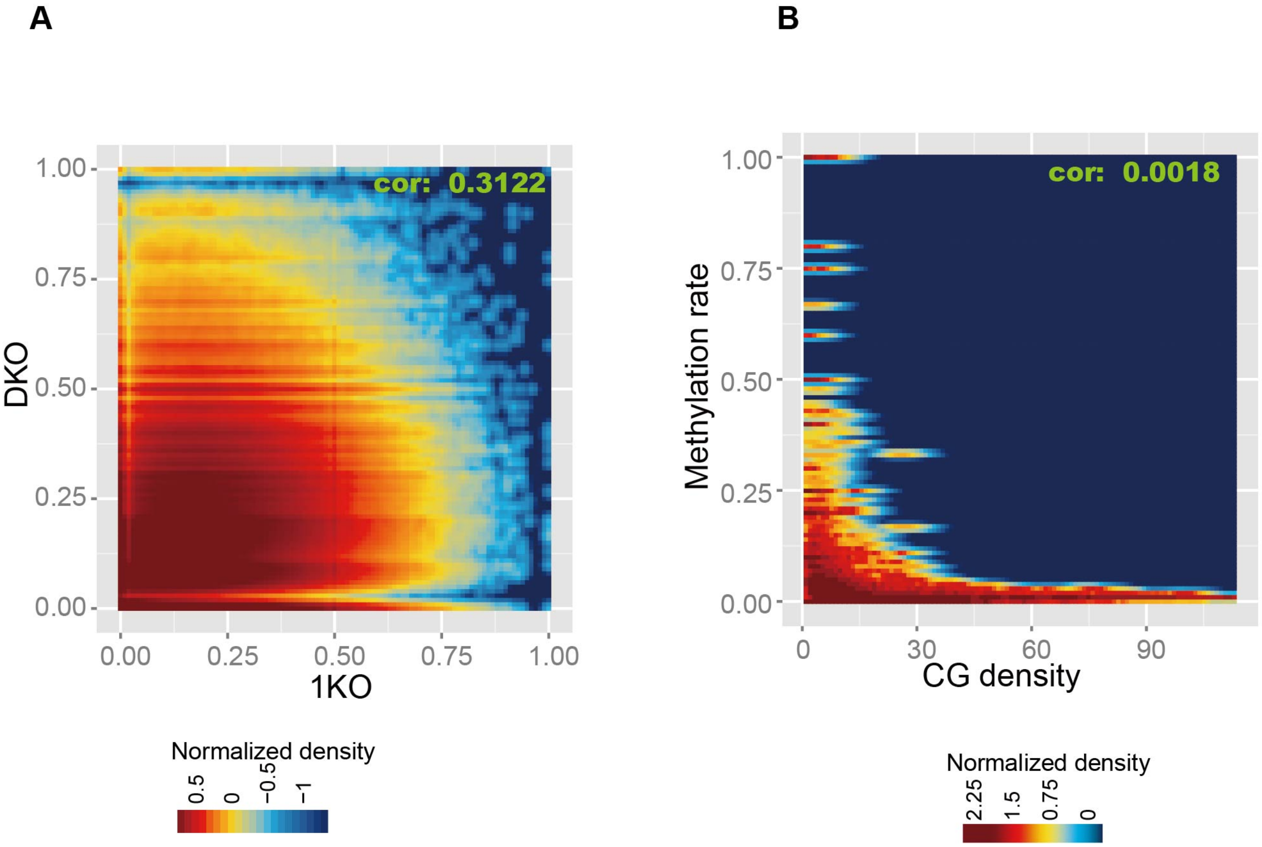

Figure S5. Methylation in DNMT-knockout cells. (A) Comparison of methylation in 1KO and DKO cells. CG sites with coverage >= 10 in both cells were used. Color represents 2D-transformed density. (B) Correlation of methylation with CG density in TKO. CG density is the number of CG sites within 600bp sliding window. Cor: correlation coefficient calculated by pearson method.

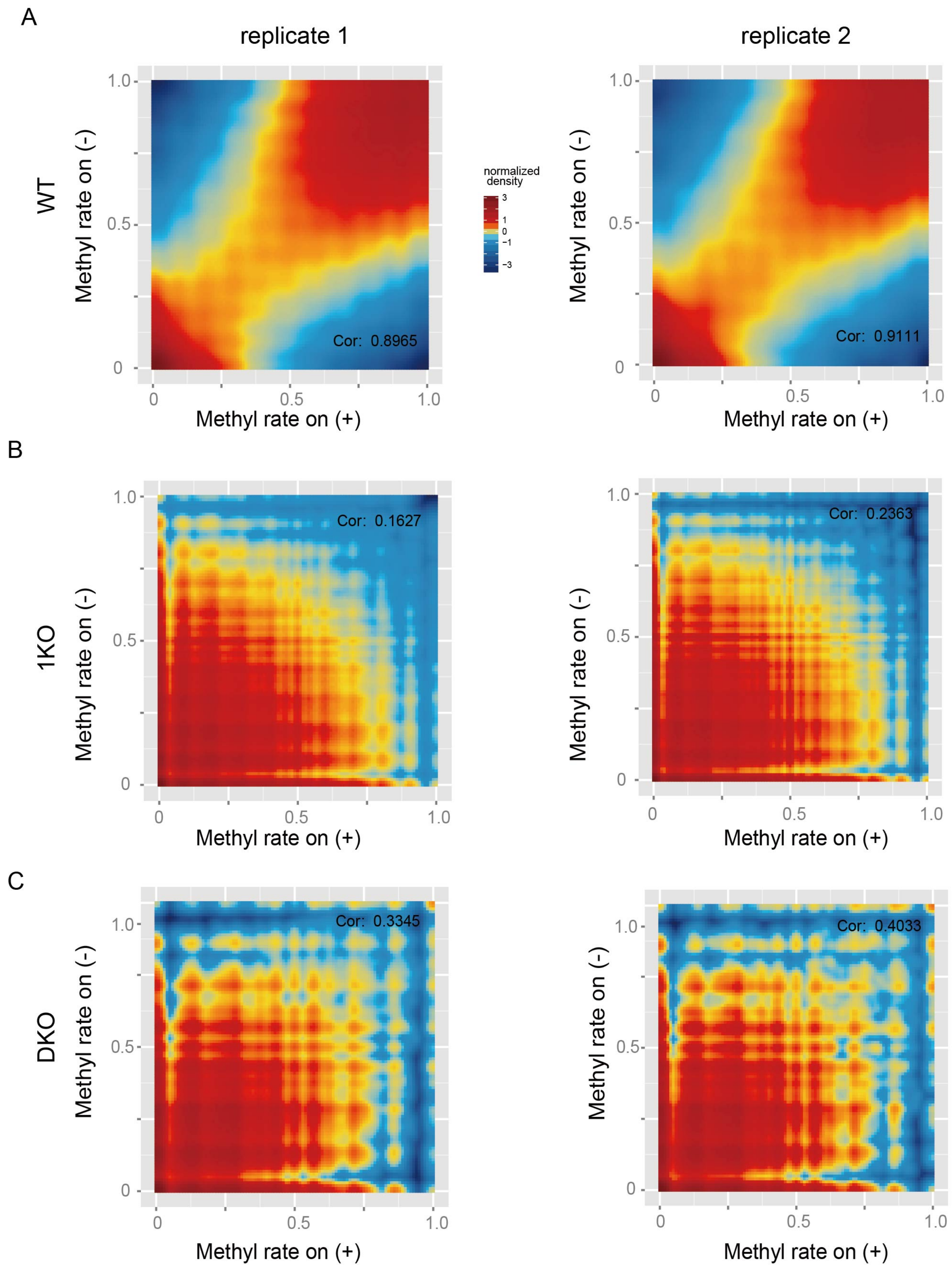

Figure S6. Correlation of CG methylation on two DNA strands is reproducible in all 3 cells. BS-seq reads that were used to generate Figure 2a-c were divided into two replicates with each replicate representing independent cell culturing, DNA sonicating, and library constructing. All the CG sites with coverage >=10 in WT and 1KO or >=7 in DKO were used.

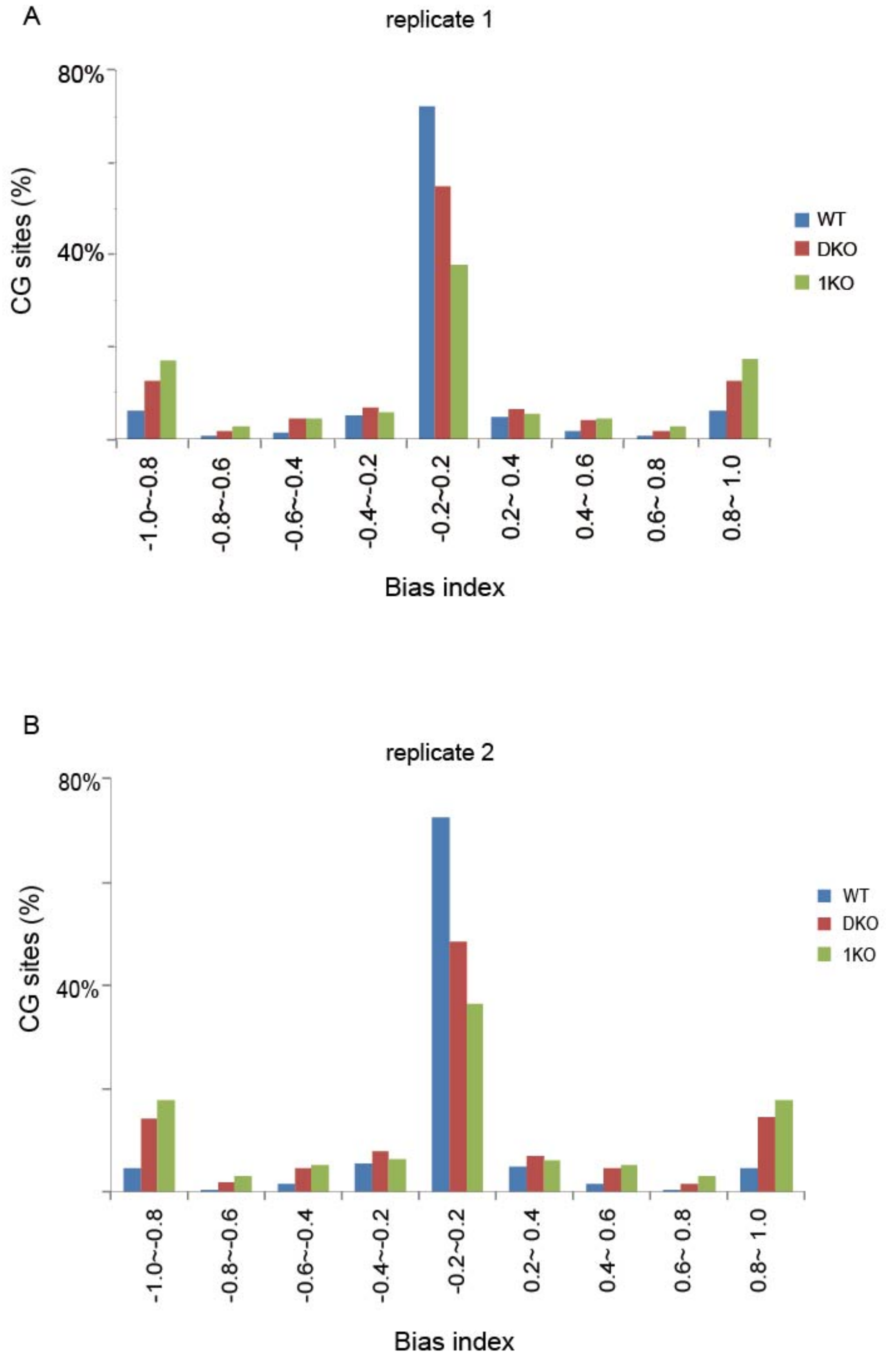

Figure S7. Distribution of bias index is reproducible in all 3 cells. BS-seq reads that were used to generate Figure 2d were divided into two replicates with each replicate representing independent cell culturing, DNA sonicating, and library constructing. All the CG sites with coverage >= 5 were used.

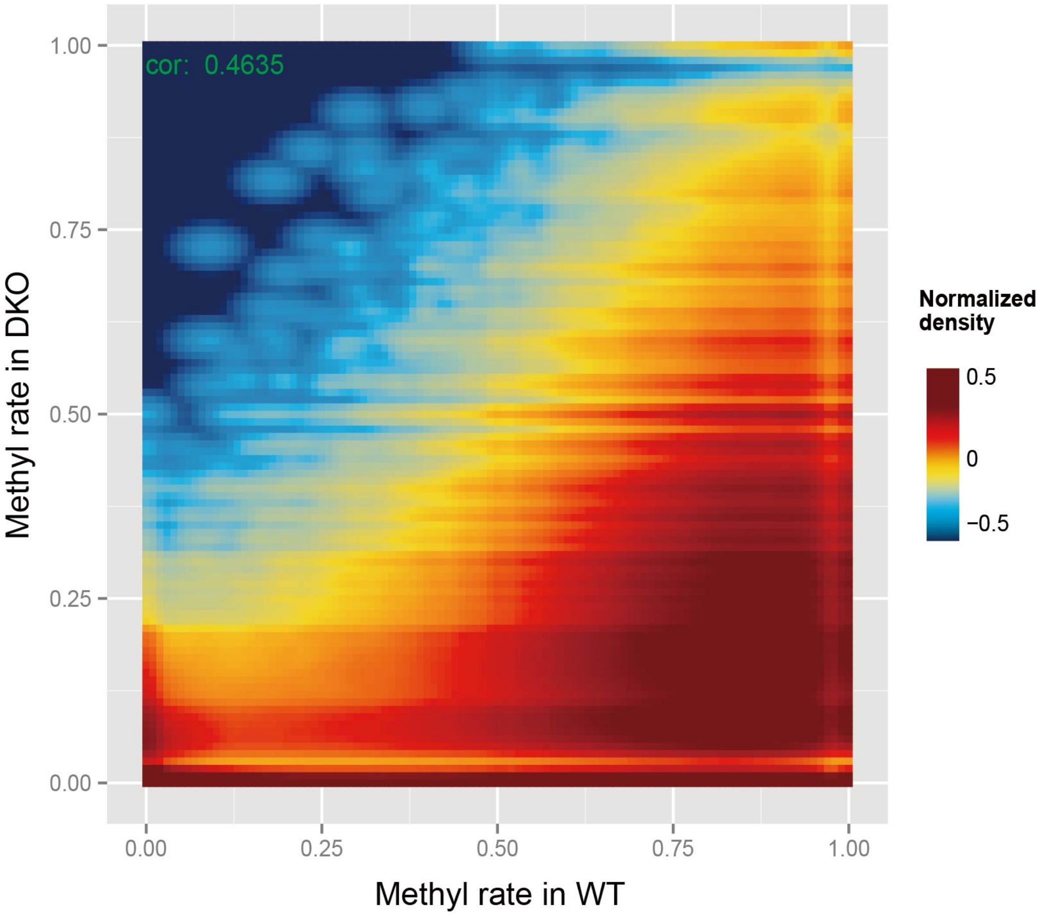

Figure S8. Correlation of methylation in DKO and WT. CG sites with coverage >= 10 in both cells were used. Color represents 2D-transformed density. Cor: correlation coefficient calculated by pearson method.

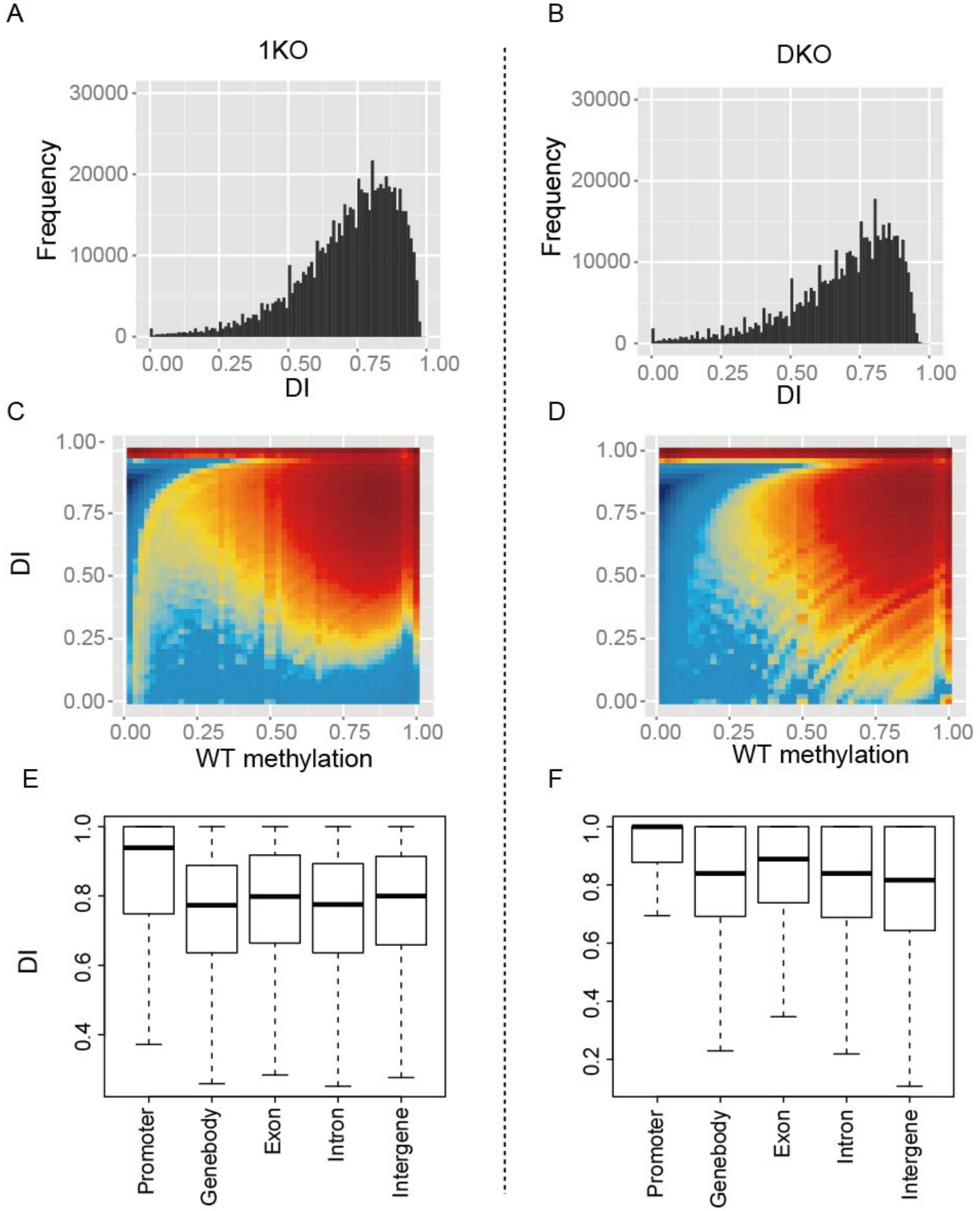

Figure S9. Deletion index. (A-B) Histogram of deletion index (DI) in 1KO and DKO. (C-D) DI show positive correlation with methylation level in WT. (E-F) DI in different gene-related genomic regions.

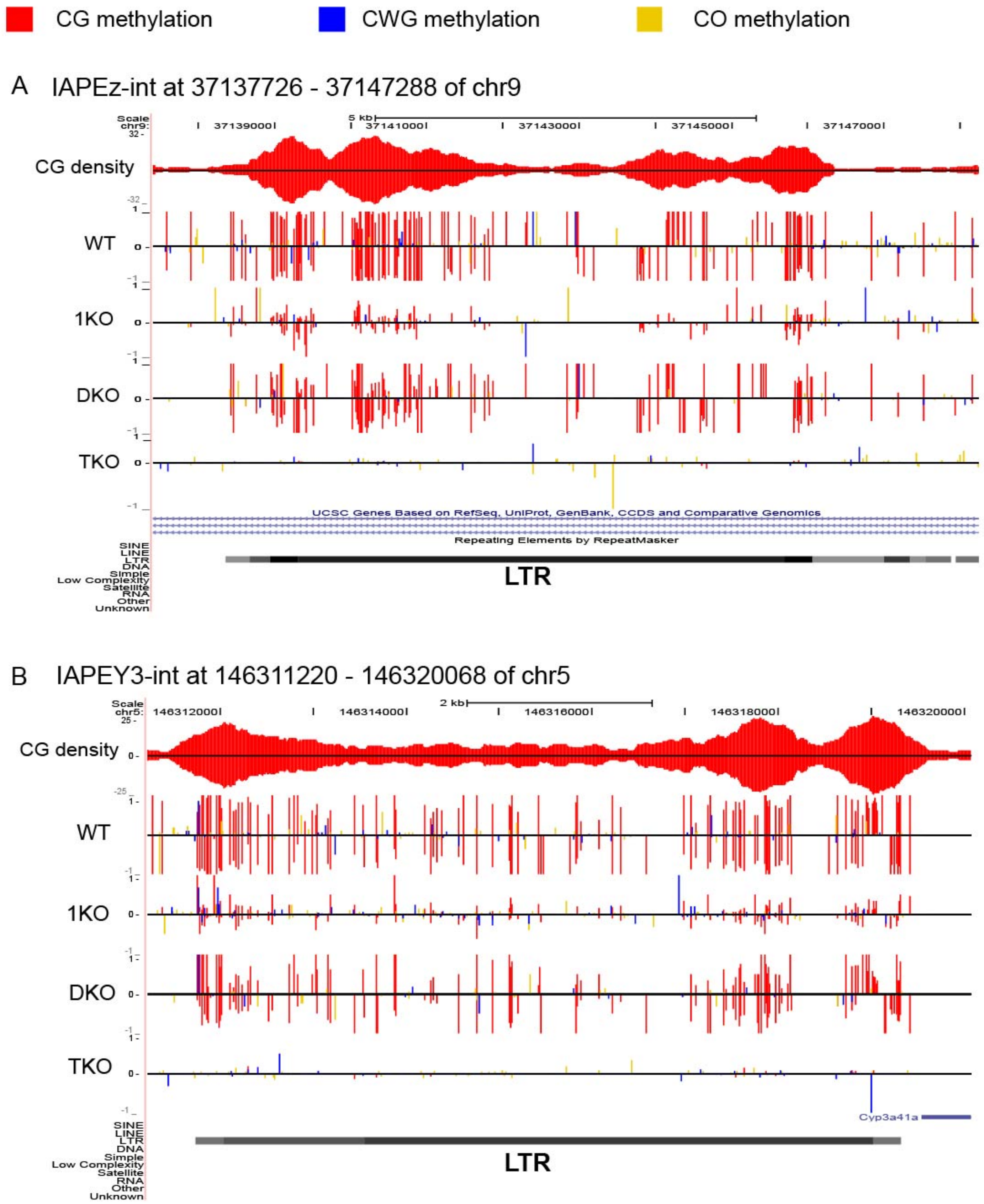

Figure S10. Screen shot of genome browser to show examples of LTR methylation in four cells. "W" in CWG refers to nucleobase A or T. CO means the C contexts that are not CG or CWG, equivalent to CHH (H = A, C, T) plus CCG.

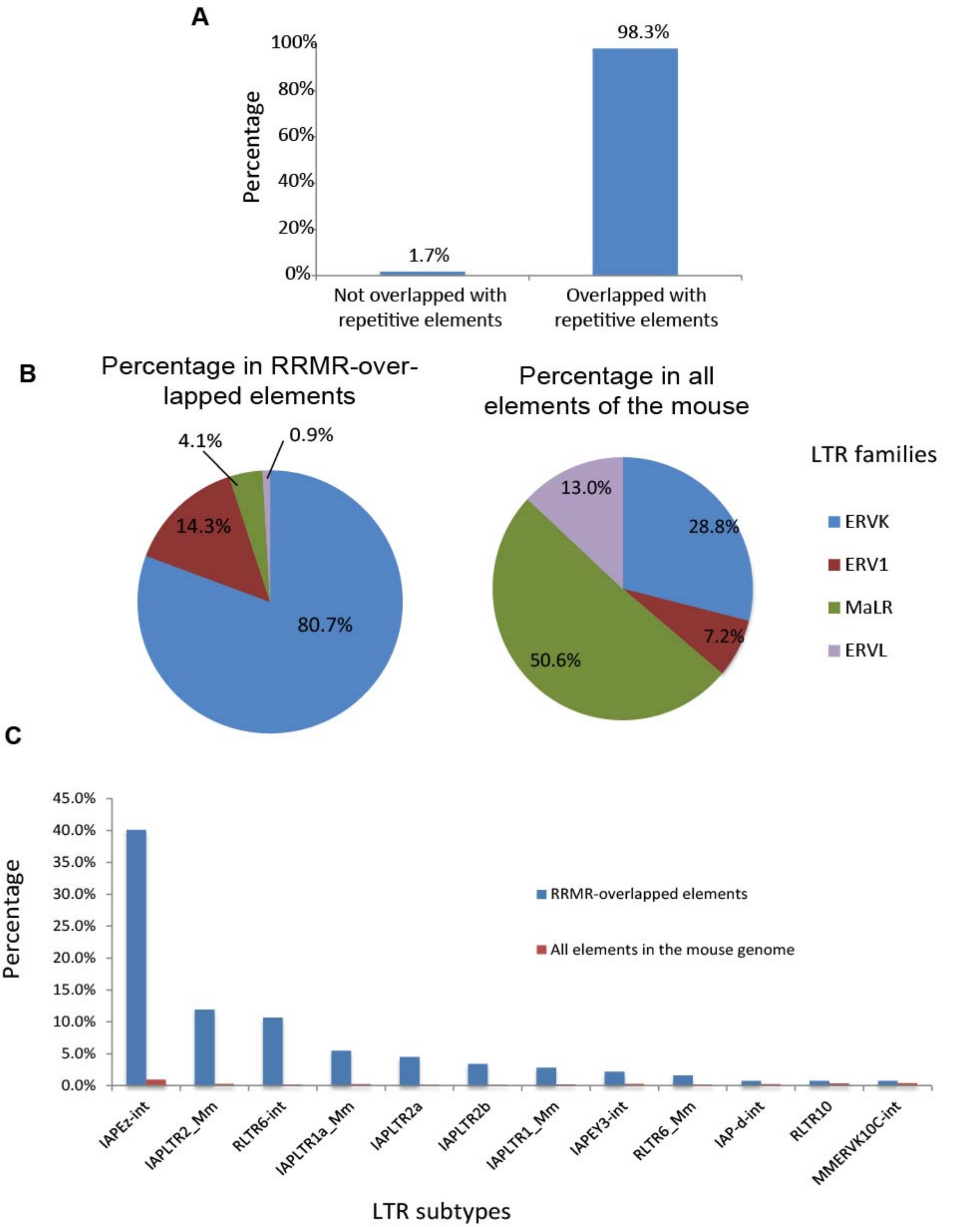

Figure S11. RRMRs are mainly located within LTRs in DKO. (A) Percentage of RRMRs overlapping or not overlapping with repetitive elements. (B) The overlapping of RRMRs with LTR families. (C) Top 12 LTR subtypes overlapped with RRMRs. Percentage of each subtype in RRMR-overlapped elements or in all mouse elements are shown.

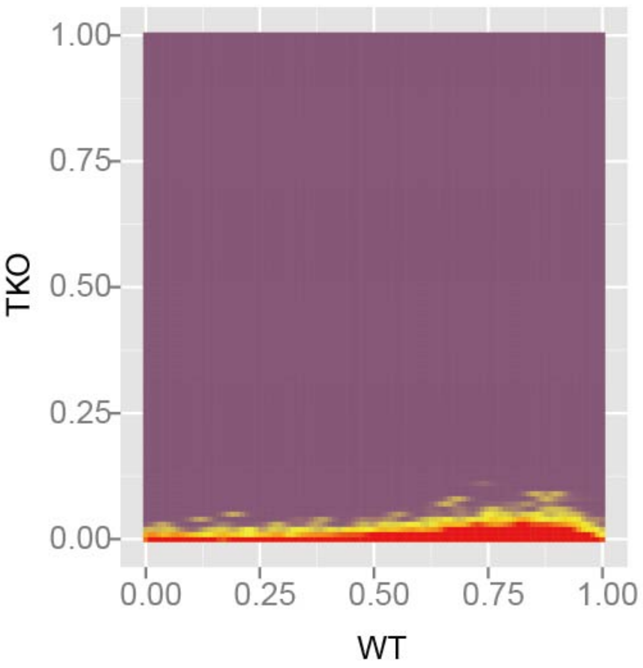

Figure S12. Correlation of LTR methylation between TKO and WT.

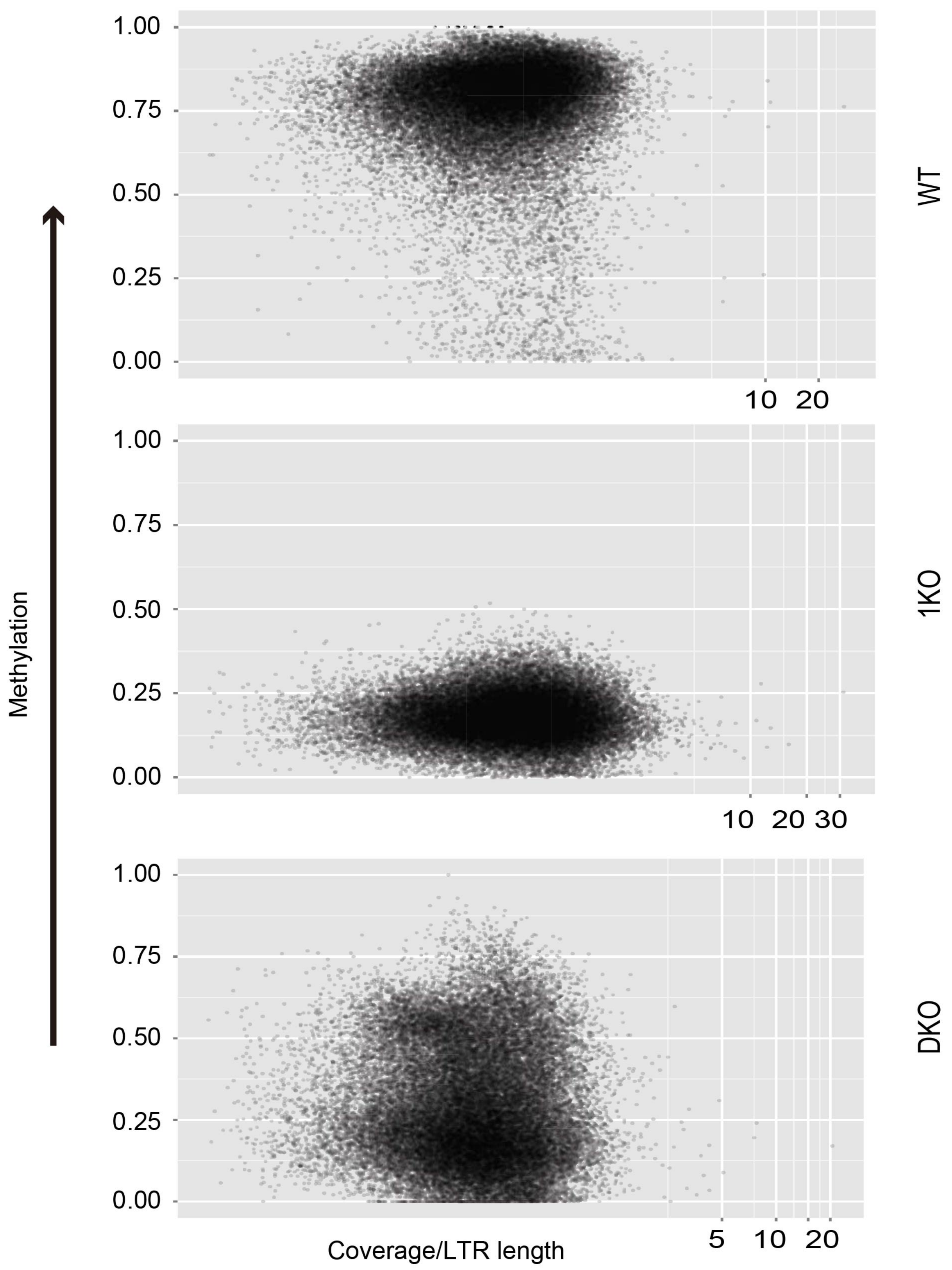

Figure S13. Methylation levels are not affected by coverage. X axis is normalized coverage that was calculated as the number of tags hitting a LTR divided by LTR length, and shown in logarithmic scale. Y axis is methylation score. The seperation of LTR into two groups in DKO is not affected by coverage.

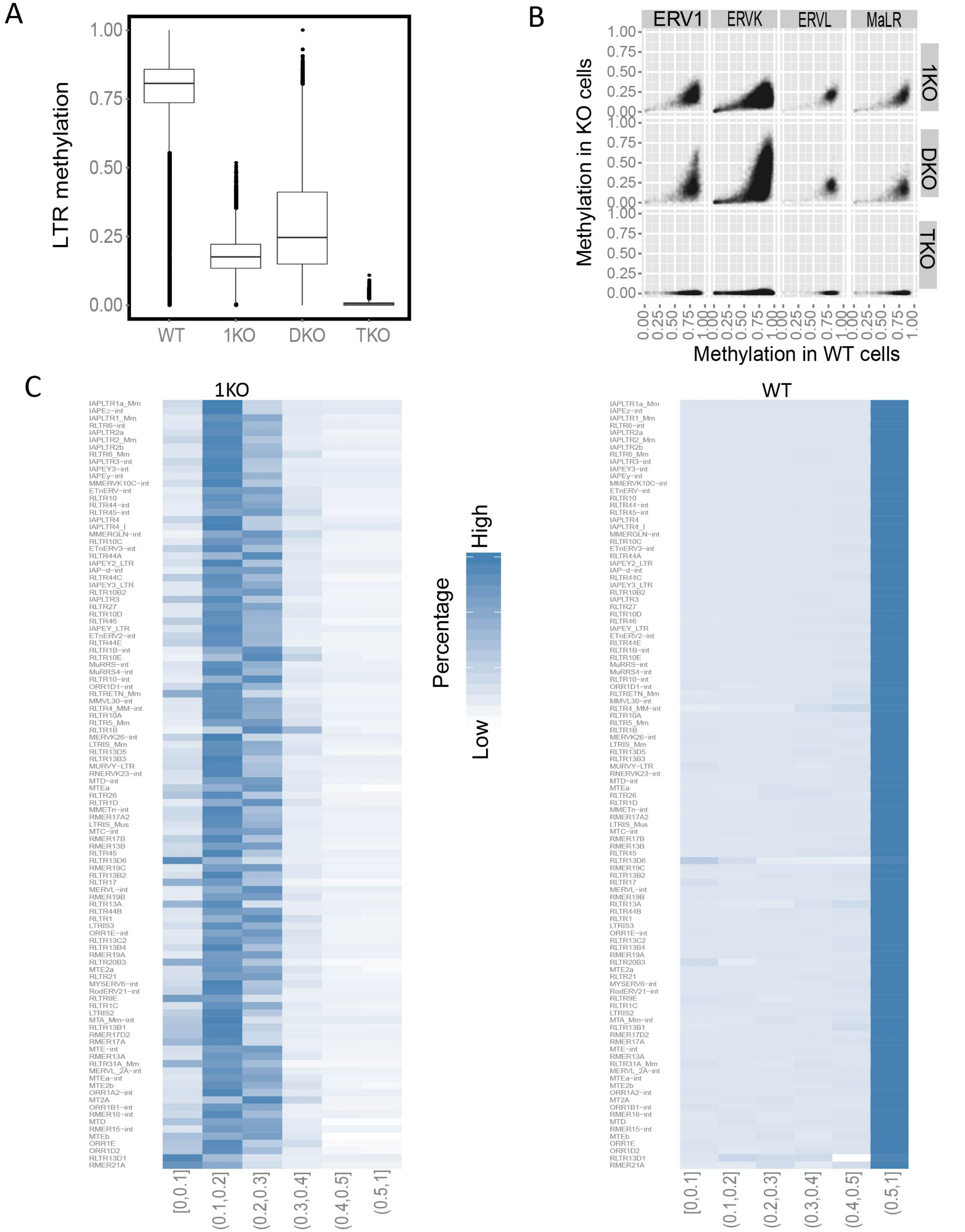

Figure S14. LTR methylation in four cells. (A) The methylation in four cells of LTRs after filtering the ones with too short length or with too few CG sites. (B) The correlation of LTR methylation in three KO cells with WT cells under each family. (C) Methylation of members in each LTR subtype in 1KO and WT cells. The subtypes have the same order as Fig 3j.

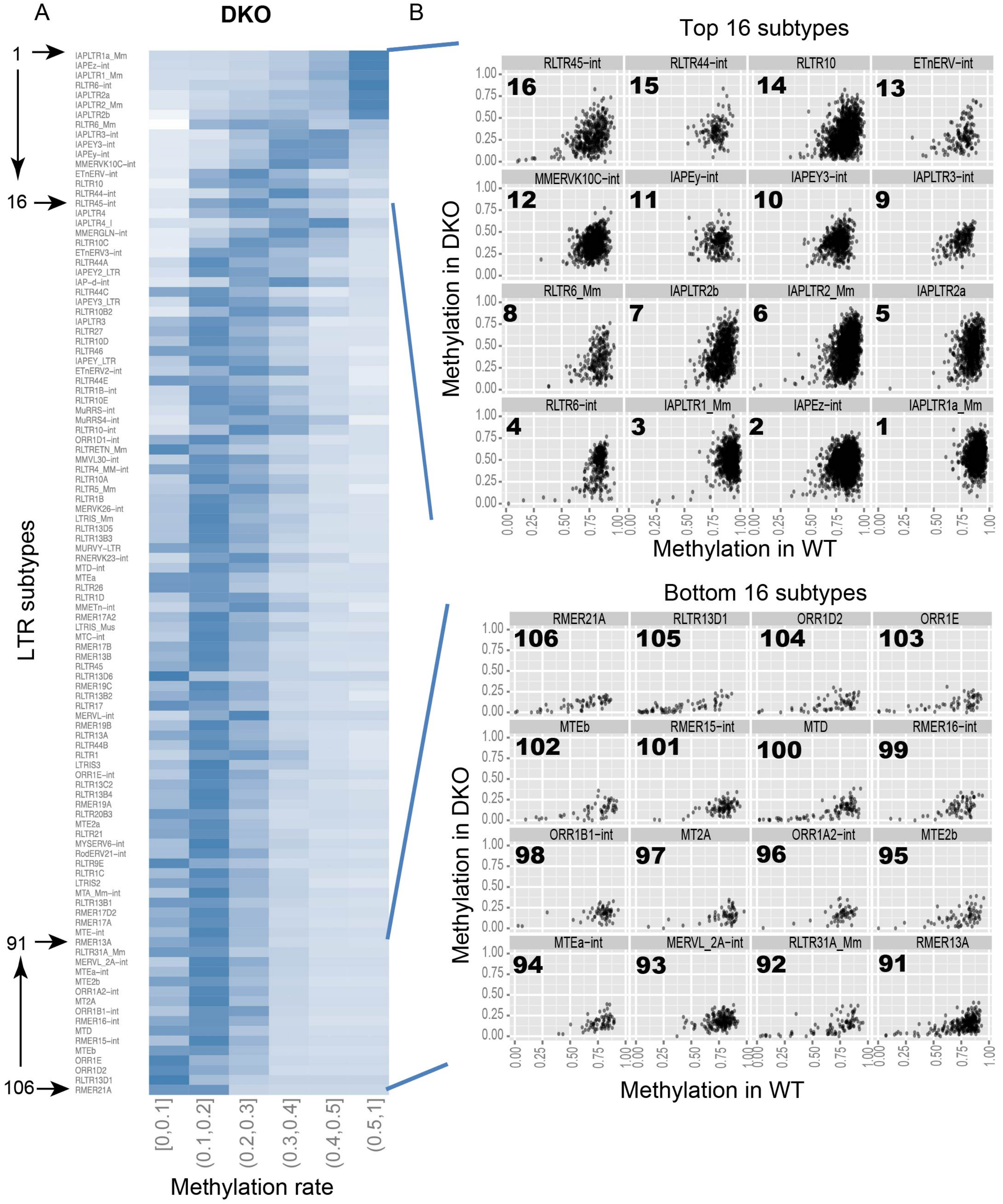

Figure S15. DNMT1 shows varying ability to retain methylation of different LTR subtypes. (A) LTR subtype ordered by overall methylation score in DKO. (B) Methylation of DKO compared to WT for the 16 top or bottom LTR subtypes.

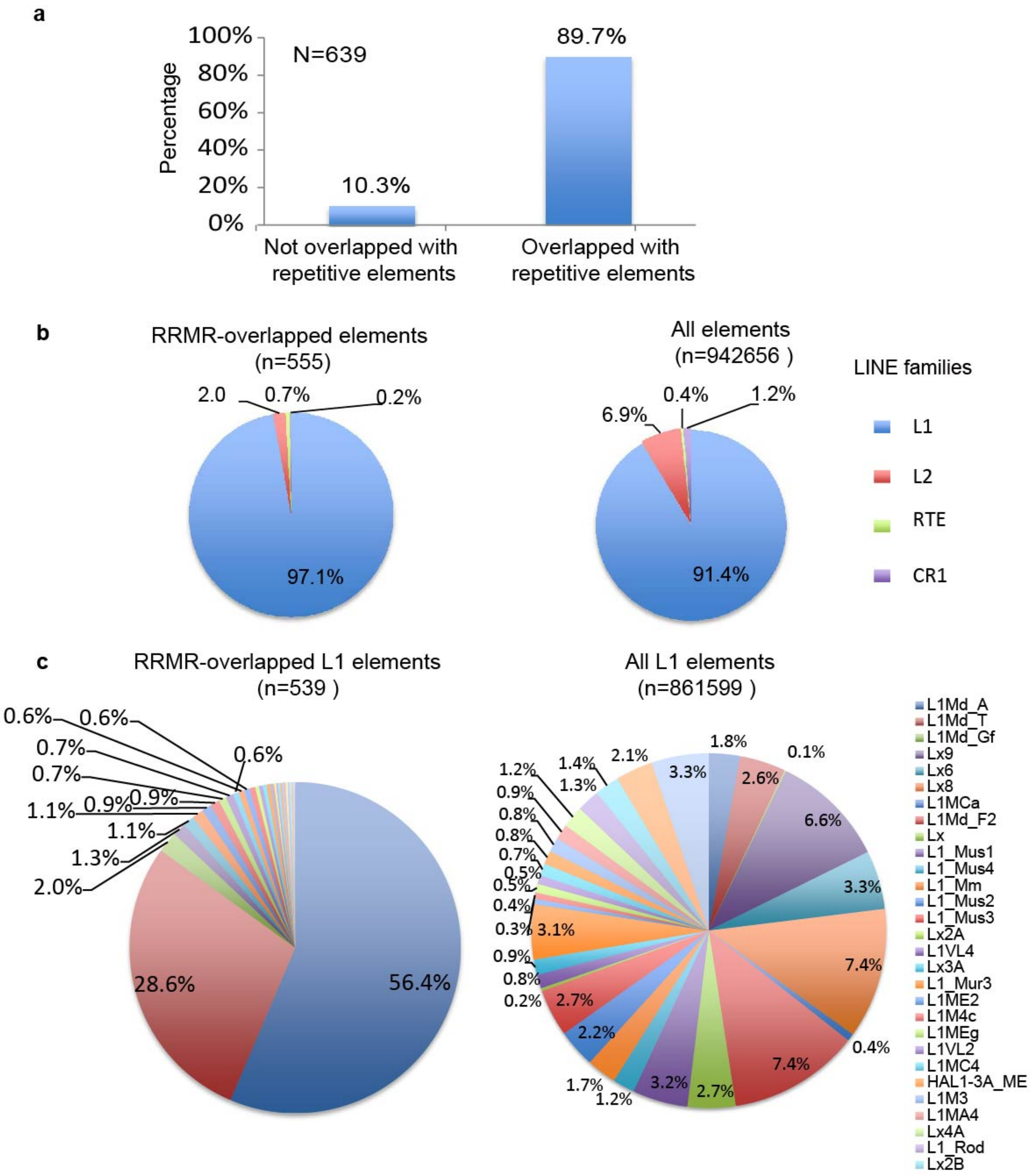

Figure S16. RRMR are mostly located within LINE in 1KO. (a) Percentage of RRMRs overlapping or not overlapping with repetitive elements in 1KO. (b) The overlapping of RRMR with LINE families. (c) The overlapping of RRMR with LINE subtypes under L1 family.

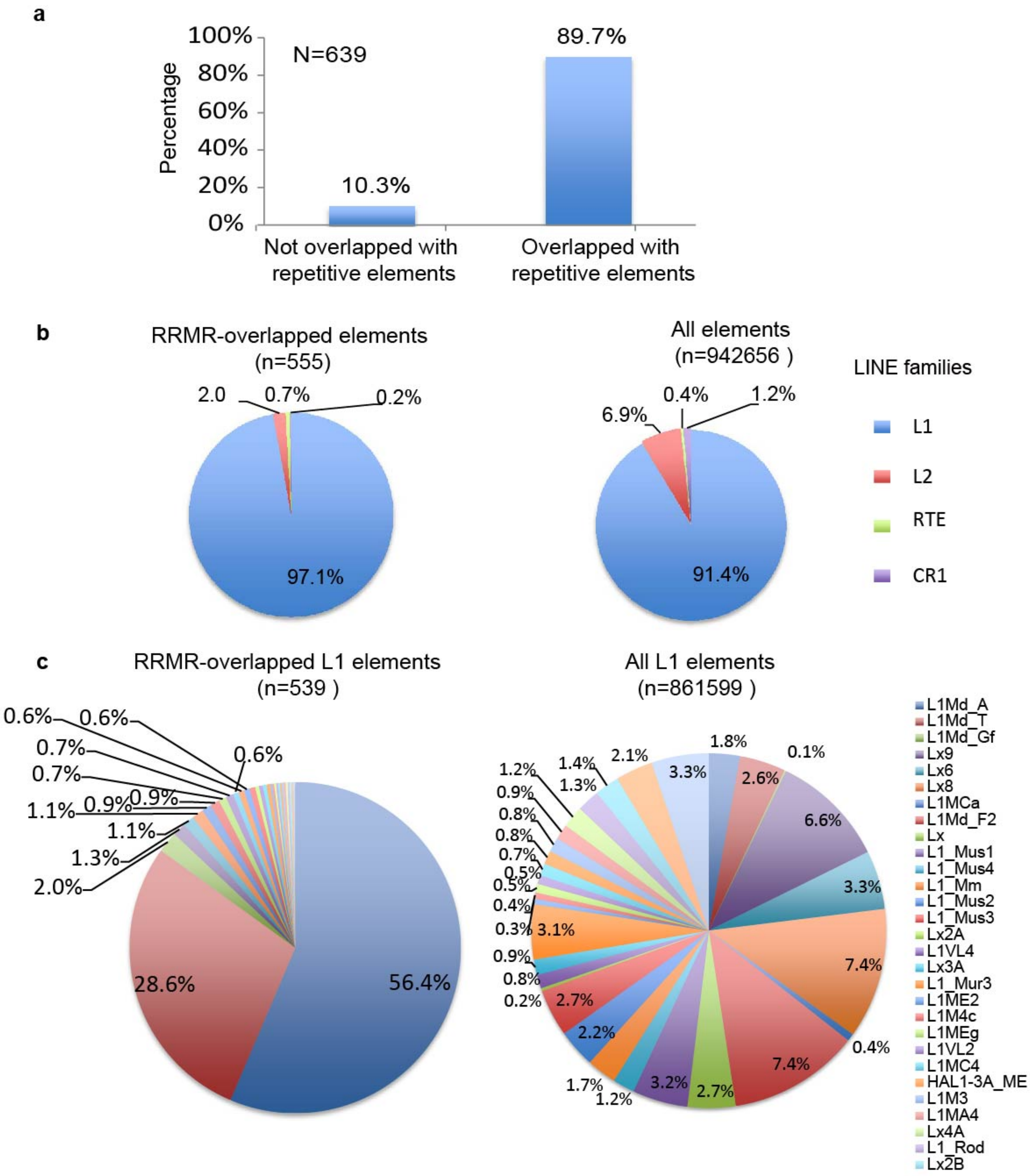

Supplementary Figure 16. RRMR are mostly located within LINE in 1KO. (a) Percentage of RRMRs overlapping or not overlapping with repetitive elements in 1KO. (b) The overlapping of RRMR with LINE families. (c) The overlapping of RRMR with LINE subtypes under L1 family.

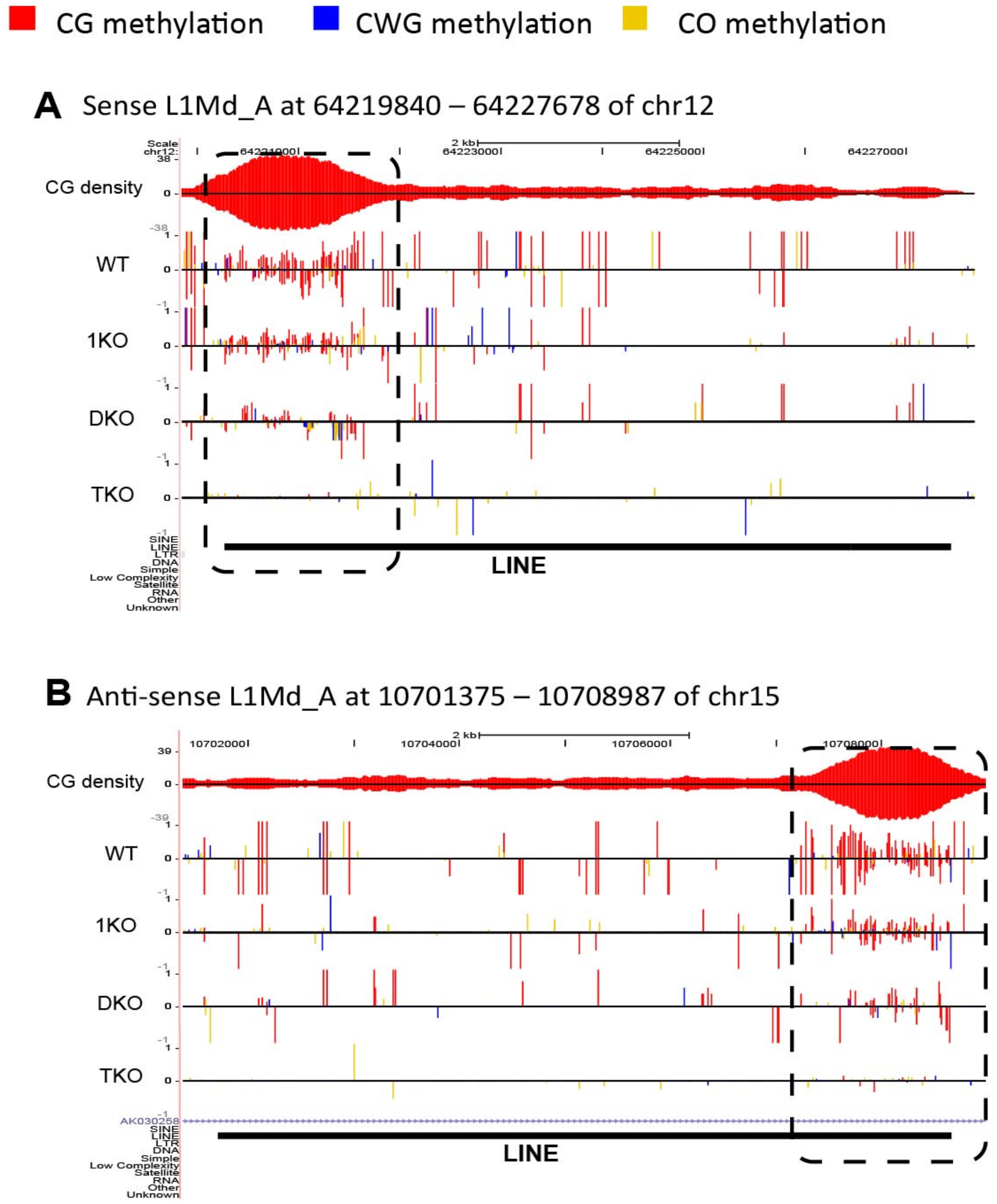

Figure S17. Screen shot of genome browser to show examples of LINE methylation in four cells. LINE promoters were marked by dashed rectangle.

A Summary of Southern blotting results from two independent investigations

RE type	WT	1KO	DKO	TKO
C-type retrovirus	★★★★★	★	★★	☆
IAP	★★★★★	★	★★★	☆

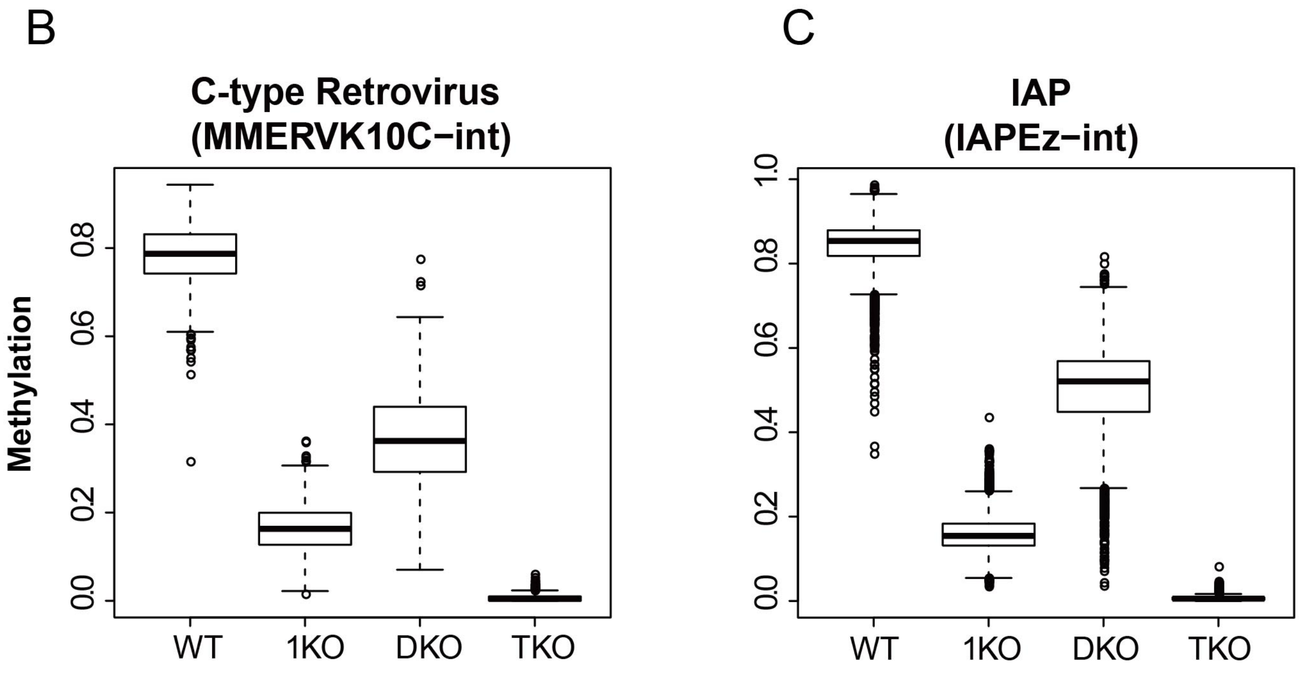

Figure S18. Data consistency with independent studies. (A) Summary of Southern blotting analysis of C-type retrovirus and IAP in WT, 1KO, DKO, and/or TKO cells conducted by other groups. Genomic DNA was digested with CpG methylation-sensitive enzyme HpaII or –insensitive enzyme MspI and hybridized with probes against corresponding elements. Methylation level was shown by number of solid stars. Open star indicates barely no methylation. The orignal data were Figure 6A-B in (Okano et al , ***Cell***, 1999, 99:247-257) or Figure 2A in (Tsumura et al, ***Genes to Cells***, 2006, 11:805-814). (B-C) BS-seq analysis of C-type retrovirus (MMERVK10C) and IAP (IAPEz) methylation in WT, 1KO, DKO, and TKO in the present study. C-type retrovirus and IAP were applied to simple filtering to remove the ones with too few CpG sites or two low coverage. Overall methylation scores were calculated for each individual member and used for boxplot drawing.

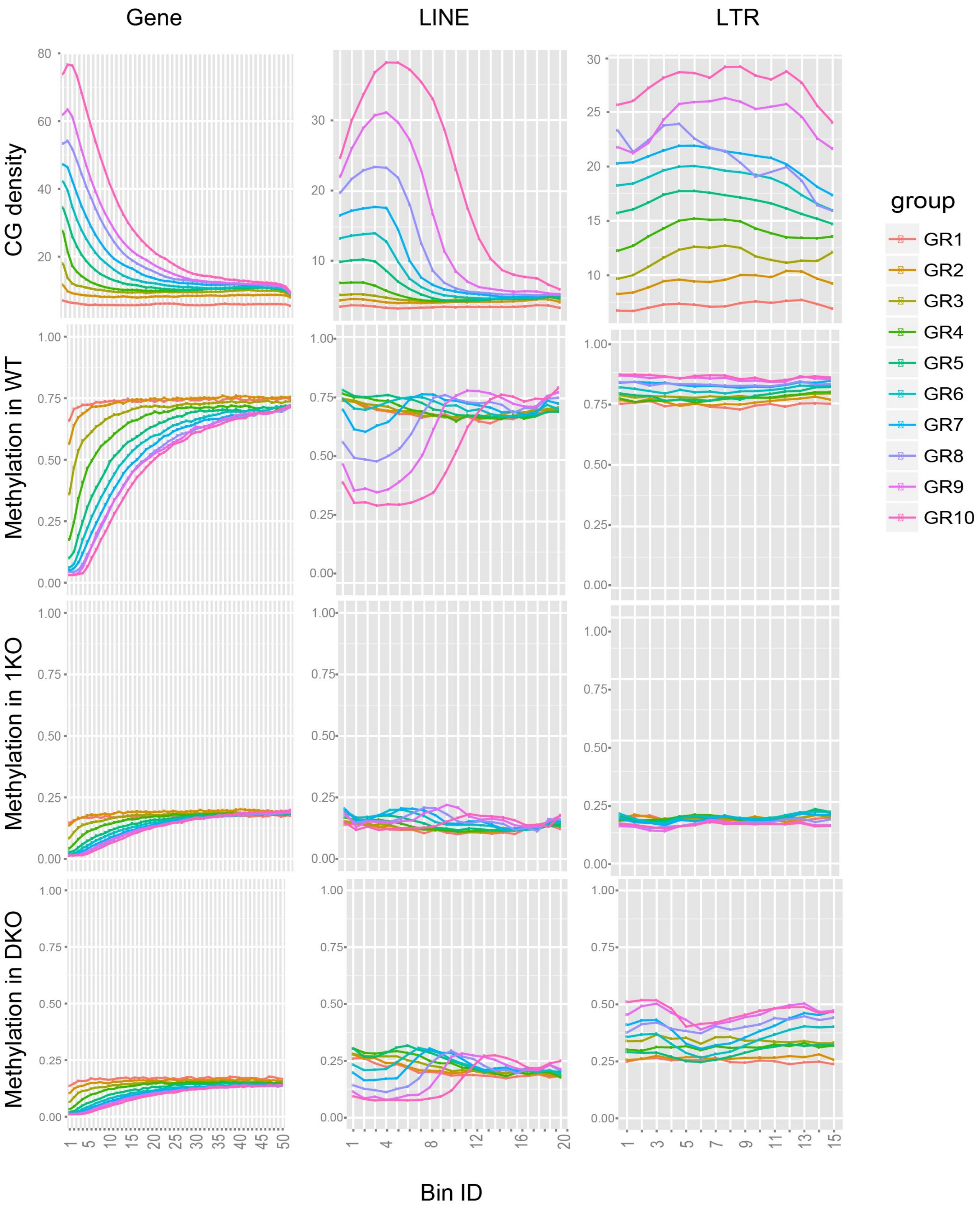

Figure S19. CG density and methylation across the body of three genomic elements. The body from TSS to TES of genes, LINEs, and LTRs was cut into 50, 20, and 15 bins, respectively, to let each bin have equal number of CG sites. Ten groups were made from each genomic element according to the height of CG peaks. CG density of each bin is the median value of the densities of all CG sites within that bin in 600bp window. Methylation rate was calculated using the overall methylation method for each bin under each group.

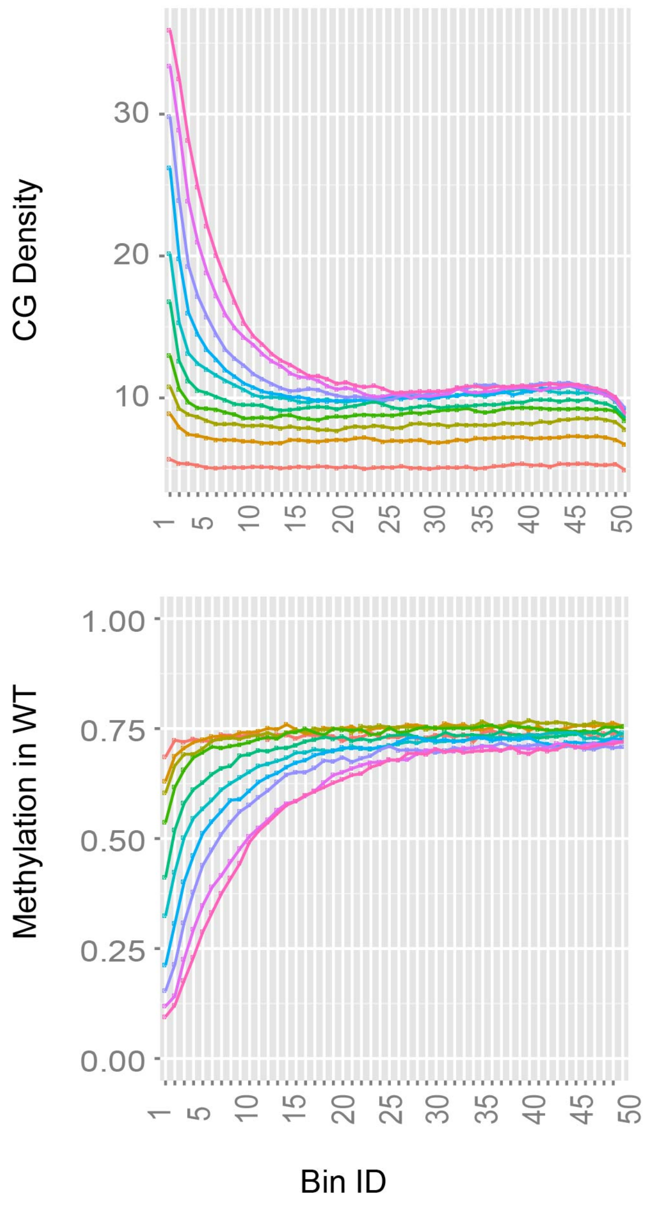

Figure S20. CG density and methylation rate for the subset of genes with CG density comparable to LINEs. Genes that have exceptional high CG density at promoter regions were filtered out and not used here.

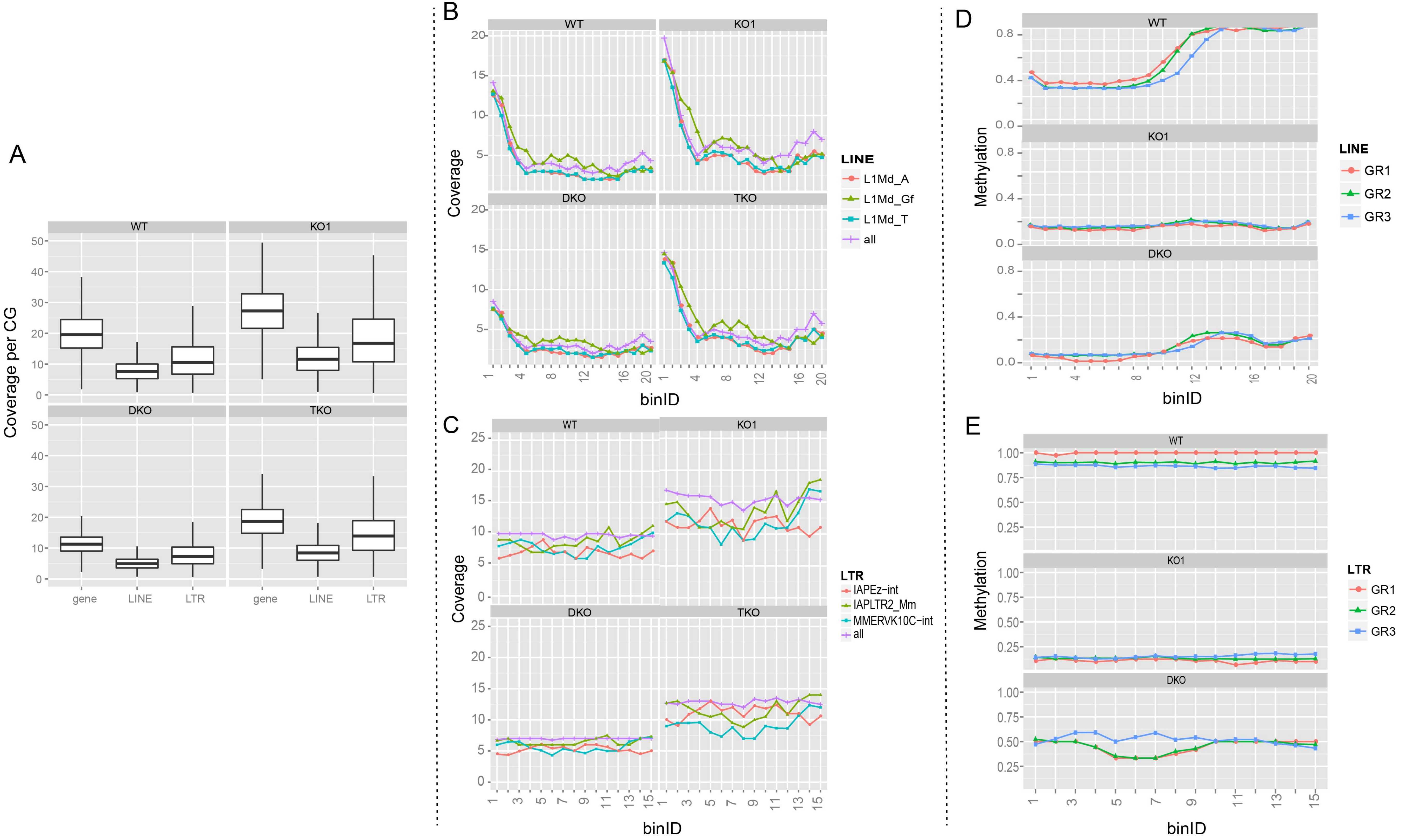
A
B
C
D
E
WT
KO1
DKO
TKO
Coverage per CG
gene
LINE
LTR
Coverage
binID
LINE
L1Md_A
L1Md_Gf
L1Md_T
all
LTR
IAPEz-int
IAPLTR2_Mm
MMERVK10C-int
all
Methylation
GR1
GR2
GR3

Figure S21. Coverage and methylation profiles in gene, LINE, or LTR. (A) Coverage per CG. The coverage on both strands of a CG site were combined together and used to make the boxplot. (B) Coverage profile on LINEs in 4 cells. LINEs from transcription start site (TSS) to transcription end site (TES) were divided into 20 equal-length bins. The median values of coverage within each bin across all the applied LINEs were calculated and used for plotting. L1Md_A, L1Md_Gf, and L1Md_T are 3 LINE subtypes with each including multiple members. "all" means all LINEs. It is obvious that the coverage goes up toward two ends with the TSS being more prominent. (C)Coverage profile on LTRs in 4 cells. LTRs from TSS to TES were divided into 15 equal-length bins. The median values of coverage within each bin across all the applied LTRs were calculated and used for plotting. IAPEz-int, IAPLTR2_Mm, and MMERVK10C-int are 3 LTR subtypes with each including multiple members. "all" means all LTRs. The coverage across LTR looks pretty flat probably because most LTRs are short and can be fully covered by use of the uniquely anchored mates. (D) The effects of coverage depth on methylation profile of LINEs in 4 cells. LINEs according to the GR10 in Supplementary Figure 19 were divided into 20 bins from TSS to TES with each bin having equal number of CG sites. These LINEs were used because they have the typical methylation pattern and are easier to detect the effects of coverage depths. These LINEs were further divided into 3 groups of GR1, GR2, and GR3 with low, medium, and high coverage depth respectively. It is obvious that even GR1 has sufficient coverage to reveal the higher promoter methylation of 1KO than DKO, as well as the typical pattern present in WT and DKO but absent in 1KO. (E) The effects of coverage depth on methylation profile of LTR in 4 cells. LTRs according to the GR10 in Supplementary Figure 19 were divided into 15 bins from TSS to TES with each bin having equal number of CG sites. These LTRs were used because they have the typical methylation pattern and are easier to detect the effects of coverage depths. These LTRs were further divided into 3 groups of GR1, GR2, and GR3 with low, medium, and high coverage respectively. It is obvious that even GR1 has sufficient coverage to reveal the higher methylation of DKO (around 50%) than 1KO (around 10%).

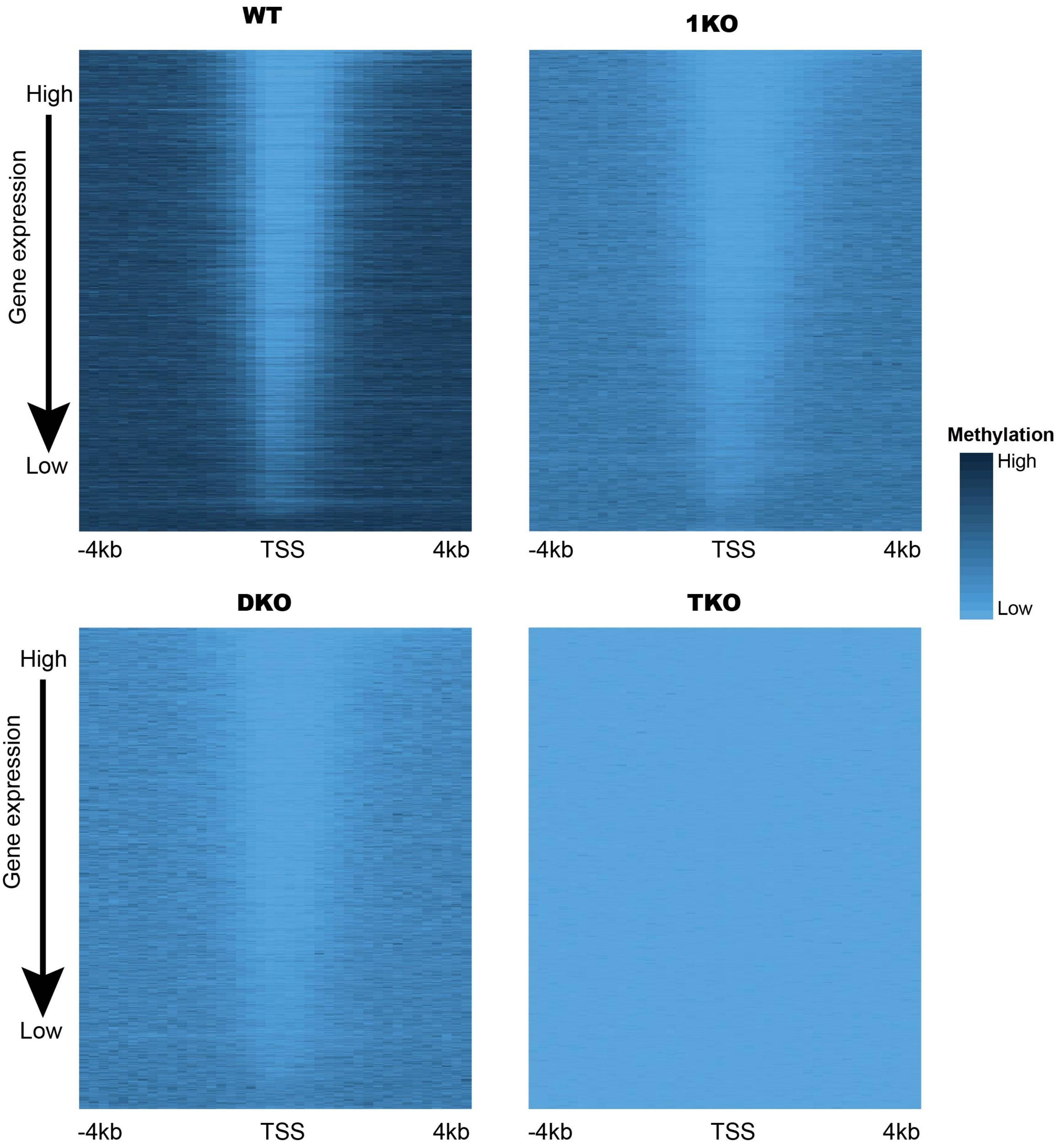

Figure S22. Gene expression is negatively correlated with methylation around TSS. Gene promoters including 4kb up- and down-stream TSS were cut into 40 equal length bins. Genes were sorted according to their expression level and cut into 500 groups with equal gene number within each group. Methylation is represented by the degree of blue color.

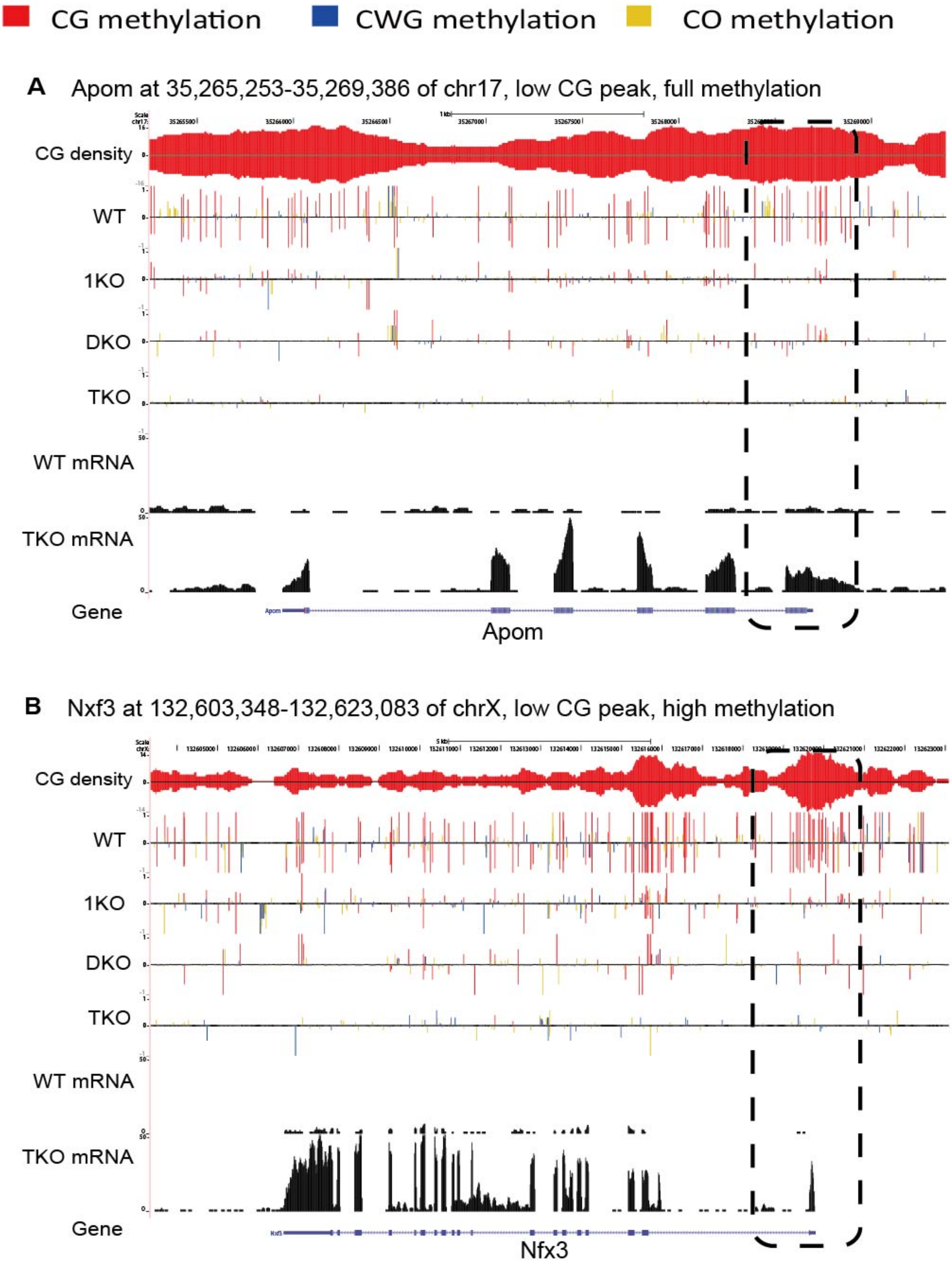

Figure S23. Genome browser screen shot of two up-regulated genes. Promoter regions are marked by dashed box.

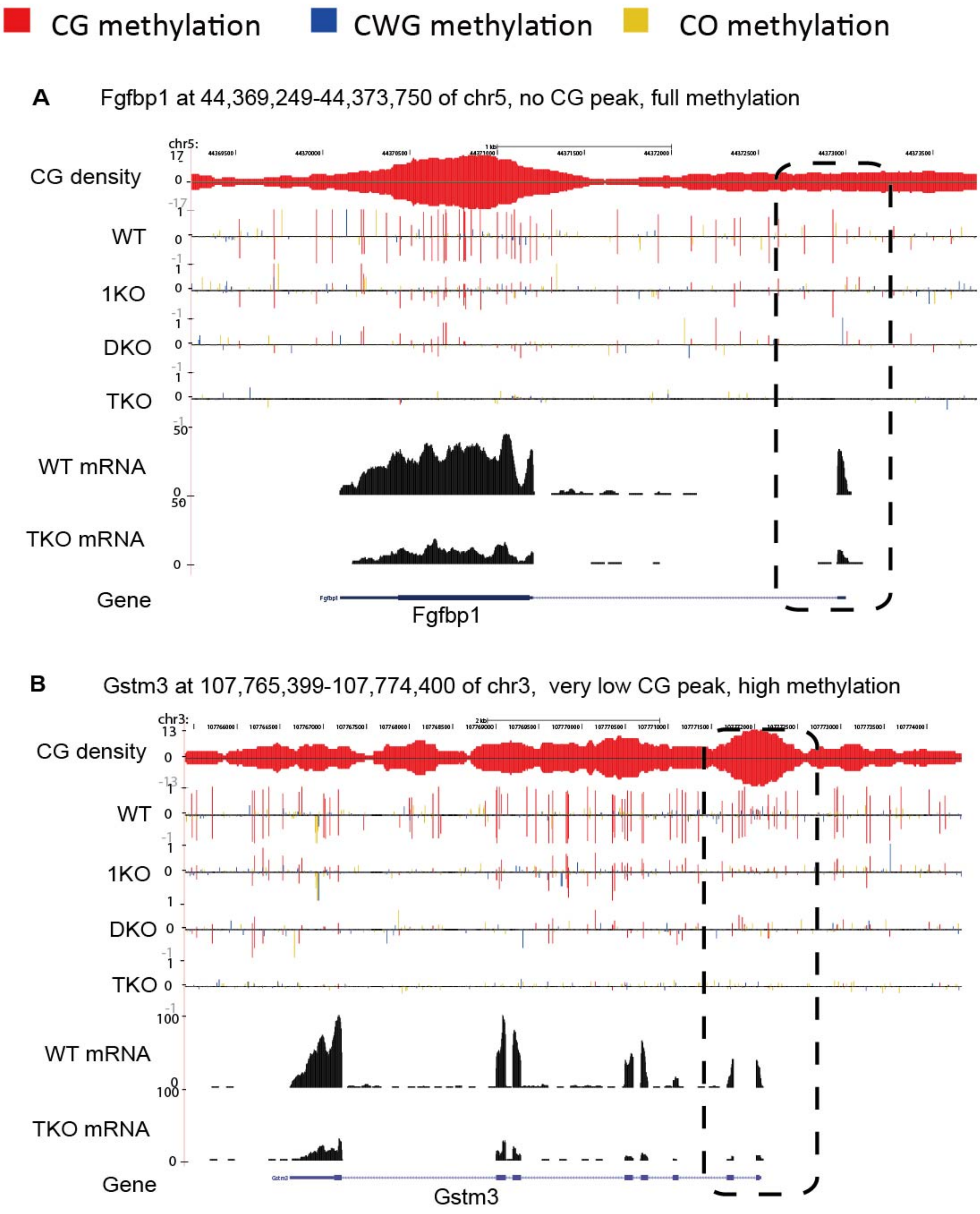

Figure S24. Genome browser screen shot of two down-regulated genes. Promoter regions are marked by dashed box.

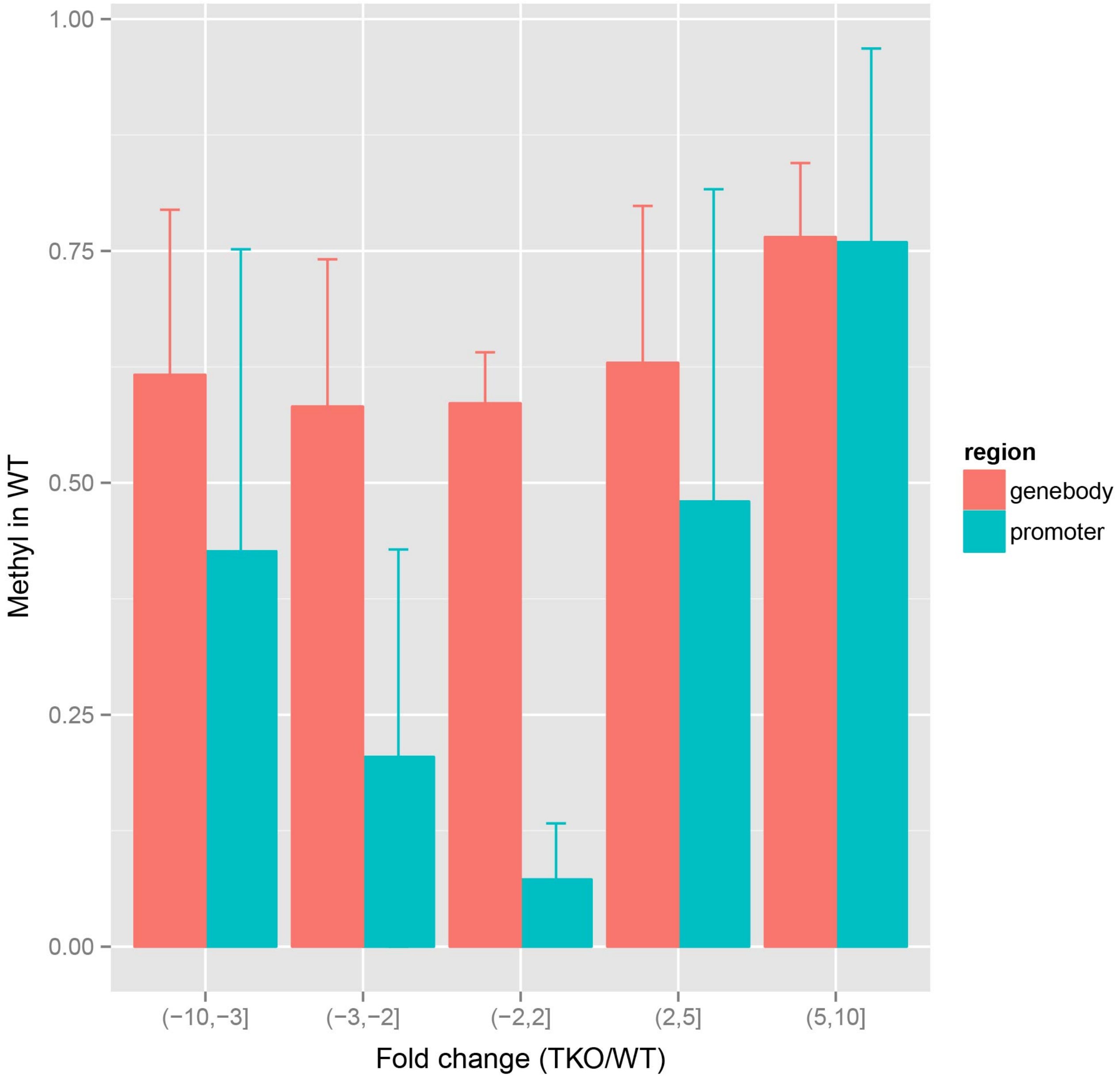

Figure S25. Correlation of gene body or promoter methylation with expression changes. Genes were divided into 5 groups according to TKO/WT expression change fold from -10 to 10, mean and standard deviation of gene body (red) or promoter (cyan) methylation in WT were calculated and shown. It can been seen that gene body keeps highly methylated across all five groups, while promoter methylation is differential with low level for unchanged genes and high level for up- or down-regulated genes.

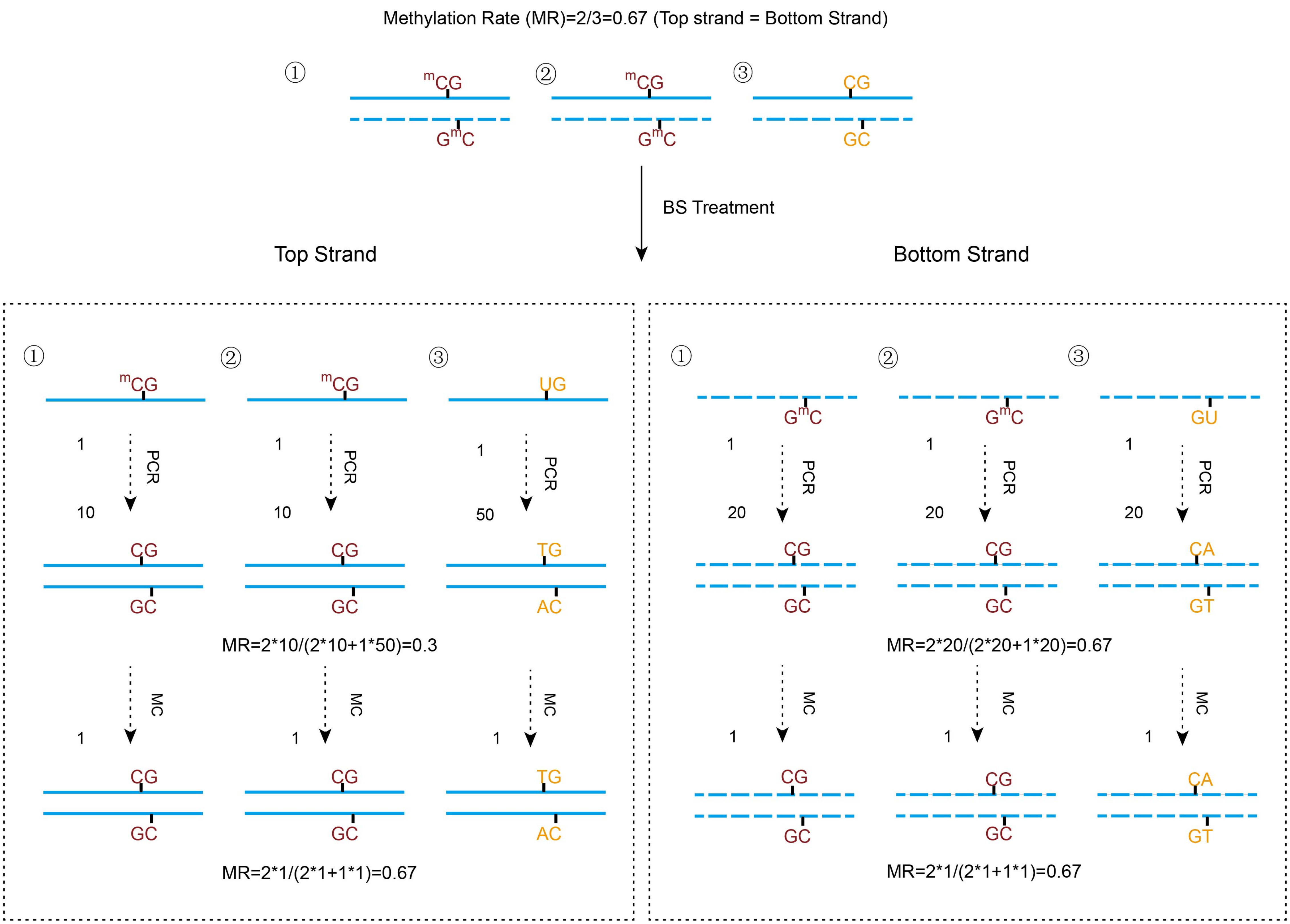

Figure S26. Moloclonization is able to effeciently get rid of the effects of PCR bias. Monoclonization is able to efficiently get rid of the effects of PCR bias. Three DNA fragments were generated from chromosomes of 3 different cells during sonication with two of them methylated and one unmethylated at indicated CG site. The actual methylation rate (MR) should be 67%. However, the MR become 0.3 on top strand after PCR because fragment 1 and 2 are amplified 10 x (from 1 molecule to 10) and fragment 3 amplified 50 x (from 1 to 50) due to PCR bias. After monoclonization, all the 10 or 50 DNA molecules are reduced to one since they map to the same chromosome positions on two ends, which let the MR become 0.67 again. For the bottom strand, since no PCR bias exists, the MR is always 0.67. The solid or dashed blue lines represent top or bottom DNA strands. MC denotes monoclonization step of data processing.
